# Efficient assembly and long-term stability of defensive microbiomes via private resources and community bistability

**DOI:** 10.1371/journal.pcbi.1007109

**Published:** 2019-05-31

**Authors:** Gergely Boza, Sarah F. Worsley, Douglas W. Yu, István Scheuring

**Affiliations:** 1 Evolutionary Systems Research Group, MTA Centre for Ecological Research, Hungarian Academy of Sciences, Tihany, Hungary; 2 International Institute for Applied Systems Analysis (IIASA), Laxenburg, Austria; 3 School of Biological Sciences, University of East Anglia, Norwich Research Park, Norwich, United Kingdom; 4 State Key Laboratory of Genetic Resources and Evolution, Kunming Institute of Zoology, Chinese Academy of Sciences, Kunming, Yunnan, China; 5 Center for Excellence in Animal Evolution and Genetics, Chinese Academy of Sciences, Kunming, Yunnan, China; 6 MTA-ELTE Theoretical Biology and Evolutionary Ecology Research Group, Hungarian Academy of Sciences, Budapest, Hungary; Santa Fe Institute, UNITED STATES

## Abstract

Understanding the mechanisms that promote the assembly and maintenance of host-beneficial microbiomes is an open problem. Empirical evidence supports the idea that animal and plant hosts can combine ‘private resources’ with the ecological phenomenon known as ‘community bistability’ to favour some microbial strains over others. We briefly review evidence showing that hosts can: (i) protect the growth of beneficial strains in an isolated habitat, (ii) use antibiotics to suppress non-beneficial, competitor strains, and (iii) provide resources that only beneficial strains are able to translate into an increased rate of growth, reproduction, or antibiotic production. We then demonstrate in a spatially explicit, individual-based model that these three mechanisms act similarly by selectively promoting the initial proliferation of preferred strains, that is, by acting as a private resource. The faster early growth of preferred strains, combined with the phenomenon of ‘community bistability,’ allows those strains to continue to dominate the microbiome even after the private resource is withdrawn or made public. This is because after a beneficial colony reaches a sufficiently large size, it can resist invasion by parasites without further private support from the host. We further explicitly model localized microbial interactions and diffusion dynamics, and we show that an intermediate level of antibiotic diffusion is the most efficient mechanism in promoting preferred strains and that there is a wide range of parameters under which hosts can promote the assembly of a self-sustaining defensive microbiome. This in turn supports the idea that hosts readily evolve to promote host-beneficial defensive microbiomes.

## Author summary

Host-associated microbiomes are complex communities, harbouring a great diversity of beneficial, neutral, or harmful species. Microbiome composition can have a significant effect on the health status and fitness of the host, and thus host species are selected to evolve mechanisms that favour the assembly of certain kinds of microbiomes over others. As we demonstrate, both by reviewing the literature and by using a detailed, spatially explicit, mathematical model, a host species can employ several cost-effective mechanisms to promote the establishment of beneficial bacteria, for example antibiotic producers, and to prevent the invasion of harmful microbes. These mechanisms include the host providing private resources in the form of a food source or inhabitable space that the preferred antibiotic-producing species are able to use to their advantage against species that are harmful to the host. After an antibiotic-producing species establishes itself with the help of the host, its colony is now self-sustaining, even if host resources now become publicly available, and can provide a reliable safeguard against parasites.

## Introduction

A growing number of studies show that microbiome composition is structured by competition [1–7], and it is hypothesized that a host could evolve to bias competition in order to promote the establishment of host-beneficial microbes [6, 8–13]. Indeed, such microbes need support because, first, it is inherently difficult to establish a colony of host-beneficial microbes in the face of competition against the huge pool of available host-neutral or host-harmful species [1, 14–17], and second, while host-beneficial microbes can produce antibiotics that are employed in direct competition against other microbes, providing protection against harmful microbes both for themselves and for the host, the cost of production can reduce host-beneficial microbial growth rates to below those of non-beneficial and parasitic microbes [18]. Here, we focus on defensive microbiomes, in which case the trait that equips the bacterial strains to be successful during the establishment of such a microbiome [9], the production of antibiotics, is essentially the same trait that benefits the host by suppressing pathogens.

We distinguish three mechanisms by which a host can selectively favour beneficial strain(s), namely by (1) providing a habitable space that the desired bacterial partner has preferential access to, (2) producing specific compounds that selectively poison undesired bacteria, and (3) providing a food resource that the desired partner is better able to metabolise. We now briefly review examples of each:

### Providing a habitable space that the desired bacterial partner has preferential access to

Vertical and pseudo-vertical transmissions fall into this category [1, 19–23]. In strict vertical transmission, host germline cells are infected with symbionts [22, 24]. Less strict transmission (‘pseudo-vertical’) is achieved by keeping non-colonised host offspring in isolation after birth until the parental microbiome can colonise it, which then shapes the composition of subsequent colonists from the environment [9, 11, 22]. In either case, the host ensures a competitor-free space for inherited microbes, which are allowed time and resources to grow on a new-born host before being exposed to competition with other colonists. For example, newly emerging *Acromyrmex* leafcutter ants are inoculated with antibiotic-producing *Pseudonocardia* bacteria within a 24-hour window after hatching [8, 25]. Mature worker ants serve as the source by carrying *Pseudonocardia* on their propleural plates, which grow to a high density around specialised exocrine glands that likely provide nutrients for bacterial growth [26, 27] (thus also serving as an example of a resource that can be metabolized by the preferred bacteria, discussed in 3. below). Similarly, female beewolf digger wasps (*Philanthus*, *Philanthinus*, *Trachypus*) inoculate their brood cell walls with a species of *Streptomyces* that they maintain in their antennal glands [28–30]. These bacteria become directly incorporated into the larval cocoon, where they dominate and produce an array of antibiotics that protect the developing larva against infection [29–31]. Analogous to the above examples, the agricultural process of applying bacteria, such as antibiotic-producing *Pseudomonas* and nitrogen-fixing *Rhizobia*, to crop seeds before sowing mimics pseudo-vertical-transmission, by ensuring that high densities of beneficial bacteria have better access to root exudates and are favoured during establishment on the plant [32, 33]. Priority effects have also been demonstrated for mycorrhizae [34], bees [35–38], wasps [28], leafcutter ants [25, 39], birds [40], plants [41], and humans [42]. A unique structure for symbiont transmission, called a ‘‘symbiont capsule,” which serves as a private space and resource, has been described for the stinkbug *Megacopta punctatissima* [43–45]. These capsules are deposited next to the eggs and provide food and protection for the symbionts until the hatchlings open the capsules and ingest the symbionts [44, 45].

### Producing specific compounds that selectively poison undesired bacteria, whilst allowing desired strains to grow

A wide range of plant species secrete compounds, known as allelochemicals, which are toxic to a broad range of bacteria, fungi, and invertebrates in the rhizosphere, as well as toward other plants growing nearby [46–49]. For example, the compound 2,4-dihydroxy-7-methoxy-1,4-benzoxazin-3-one (DIMBOA) is an antimicrobial produced by maize seedlings [48], which the plant-beneficial species *Pseudomonas putida* is able to degrade, thus avoiding its effects. *P*. *putida* also uses this compound as a chemoattractant and a signal for upregulating the production of the broad-spectrum antibiotic phenazine [48]. Together, these mechanisms allow *P*. *putida* to colonise maize roots in the presence of mostly DIMBOA-intolerant, competitor bacteria [48]. Similarly, the rhizobial species, *Mesorhizobium tianshanense*, which forms root nodules on liquorice plants, is able to outcompete other bacteria in the rhizosphere due to an efflux mechanism that confers resistance to the antimicrobial compound canavanine. Canavanine is abundant in liquorice root exudates and thus allows the host to filter out non-beneficial rhizobial species [50]. As another example, nitric oxide (NO), a potent oxidising agent and antimicrobial, can play an important role in dictating symbiont specificity [51, 52]. A classic example arises during the symbiosis between the bobtail squid, *Euprymna scolopes*, and bioluminescent bacteria in the species *Vibrio fischeri*. *V*. *fischeri* are the exclusive colonisers of the squid’s light organ, where they emit light to deceive predators, and are acquired horizontally from the environment within 48 hours after squid hatching [53]. High nitric-oxide synthase (NOS) activity and its product NO can be detected in the epithelial mucus of the light organ during the early stages of bacterial colonisation [54], which *V*. *fischeri* are able to tolerate via the activity of two proteins, flavohemoglobin (*Hmp*) and a heme NO/oxygen-binding protein (H-NOX) [55–58]. Eliminating the genes for these proteins in *V*. *fischeri* leads to colonisation deficiency [56, 58], and diminishing the concentration of host NO results in a greater diversity of non-mutualistic bacterial species in the light organ epithelium [54]. Similar mechanisms of host selection are also reported for other animal species. For example, members of the *Hydra* family produce antibacterial arminins that help them to shape the establishment of the bacterial microbiota during their embryogenesis [59]. *Hydra* not only suppresses undesired strains [59] but also modifies the quorum-sensing signals by which bacteria communicate, hence manipulating the social behaviour of bacteria [60].

### Providing a food resource that the desired partner is better able to metabolise

Enhanced metabolic activity from consuming a private resource can confer competitive superiority on a preferred microbial strain. Besides acquiring higher reproduction and growth rates, the beneficial bacteria can also achieve a higher rate of antibiotic production that results in the suppression of competitors [61] or achieve a higher production of other factors that promote colonization and symbiotic interaction with the host, such as adhesive molecules facilitating biofilm formation on the host surface [62, 63]. The provision of specific metabolites is thought to play a key role in structuring the species-specific microbial communities associated with marine corals [64, 65]. Coral juveniles, as well as their dinoflagellate symbionts, produce large quantities of the compound dimethylsulfoniopropionate (DMSP) [66]. *In vitro* and metagenomic studies have shown that several coral-associated bacterial groups can specifically metabolise the DMSP and use it as a sole carbon and sulphur source [64, 65, 67]. Such species are also amongst the first bacteria to colonise coral larvae, suggesting a nutritional advantage for them over bacteria that cannot degrade DMSP [64, 68]. This includes a species of *Pseudovibrio* which can additionally use DMSP as a precursor for the production of antibiotics that inhibit coral pathogens [65]. Another example of a specific host-derived resource is human breast milk, which contains a large number of complex oligosaccharides that are preferentially consumed by a single species of co-adapted gut bacterium *Bifidobacterium longum* subsp. *infantis* [69]. In plants, experiments have shown that root exudates can be directly metabolised by the microorganisms that live endophytically within the plant roots [70–73]. Different species exude different groups of metabolites, and studies suggest that plant hosts may be able to tailor root exudate composition in order to recruit bacteria with particular metabolic traits [46, 70, 73]. For example, the concentration of the plant phytohormone salicylic acid (SA) has been shown to correlate with the abundance of several bacterial taxa, including the antibiotic-producing genus *Streptomyces* [73, 74], which can use SA as a sole carbon source [74, 75]. As discussed earlier, leaf-cutter ant exocrine glands, which provide a nutrient source for *Pseudonocardia* bacterial growth, also fall into this category [26].

These mechanisms achieve one of two effects: (I) they either ensure the protected growth of the preferred strains and/or (II) they enhance the competitive abilities of preferred strains against non-preferred strains, for example by increasing the rate of antibiotic production or the rate of growth of the beneficial strain, for certain duration of time. Taken together, these examples show that hosts have access to multiple mechanisms that can provide a ‘private resource’, in the form of space and/or food, to a subset of bacterial strains, and if those strains are beneficial to the hosts, the host is selected to apply one or more of these mechanisms to assemble host-beneficial microbiomes.

However, once the private resource is withdrawn, the host becomes a public habitat on which a diversity of microbes can thrive, either feeding on generally available resources coming from the host (for example, secretions, excretion, or dead epithelium) or from the physical environment. The question therefore is whether and how a time-limited private resource can be translated into a persistent host-beneficial microbiome.

**Fig 1:**
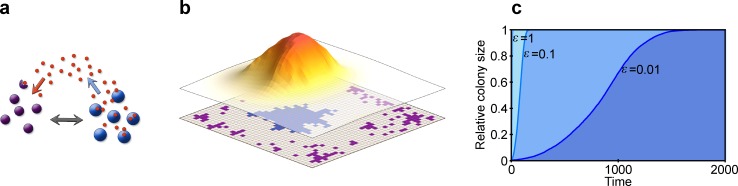
Model schematics. (**a**) We model two strain types, parasitic (violet shading) and antibiotic-producer (blue shading), which compete with each other directly (grey arrow), and indirectly via the diffusing antibiotic (red dots and coloured arrows). (**b**) The modelled *N* = M*M grid (bottom layer) represents the colonisable surface of the host, and each point in the grid can be inhabited by a single individual (coloured quadrant). The produced antibiotic (upper layer) diffuses freely on the grid, and its concentration decreases farther from the producing source (the shading and height depicting the concentration) and also decays with time. (**c**) The growth dynamic of a colony follows a logistic trend in the model. We show the relative colony size (y-axis) with respect to time (x-axis) with *ε* = 1 (light blue), *ε* = 0.1 (medium blue), and *ε* = 0.01 (dark blue), where *ε* is the fraction of randomly chosen grid cells that is updated in the cellular reproduction and death processes. The smaller the *ε*, the slower the growth in our model. Relevant model parameters are: *D* = 5, Δ*t* = 1/10, *u* = 100, for **a** *n*_B,0_ = 100, *N* = 10 000, and for **b** *n*_B,0_ = 1, *M* = 40, *ρ*= 1, *α*_B_ = 0.5, *β*_B_ = 0.6, *γ*_B_ = 0.3, *φ* = 0.5.

To answer this question, we now abstract these mechanisms into an individual-based, spatially explicit model of host-associated defensive microbiomes (Fig 1A and 1B) (reviews in 9, 29, 30), which typically contain antibiotic-producing bacteria [76, 77]. In our model, dispersal and direct competition for empty sites is limited to small numbers of neighbouring individuals, in accordance with experimental results [78]. At the same time, due to diffusion, indirect, antibiotic-mediated competition can occur amongst distant bacteria. We show that a host is indeed able to assemble a defensive microbiome, by providing a private resource that has the effect of exploiting the community bistability which emerges when bacterial species engage in interference competition [9]. We also show that the host only needs to provide the private resource until the beneficial microbe’s colony reaches a self-sustaining size, that bacterium-produced antibiotic defends the colony most effectively at an intermediate level of diffusion rate, and that the antibiotic-efflux resistance mechanism is the most efficient mechanism for achieving competitive superiority.

We focus our modelling on the community dynamics of the bacteria, and therefore we only model the host indirectly. This is because bacterial community dynamics play out much more quickly (hours to days) than does the coevolutionary response of a host lineage to the fitness consequences of its achieved microbiomes. In other words, a host might evolve a new private-resource trait that changes the trajectory of microbiome assembly, which then affects host fitness and either selects for or against that new trait. Our focus is on the first half: how differences in the host-provisioning of private resources affect microbiome assembly, which is not well understood. We also simplify the modelling by binning multiple bacterial species into two archetypes, beneficial and parasitic, because we are interested in whether (any number of) beneficial species can coexist with or even competitively exclude (any number of) parasitic species. The same approach has long been used in community ecology, such as in modelling the coexistence of pioneer vs. shade-tolerant trees and superior competitors vs. superior dispersers [e.g. 79–81]. Typically, once two types can be shown to coexist, subsequent modelling shows that the same coexistence mechanism can be extended to allow the coexistence of multiple species [e.g. 82], or additional mechanisms can be invoked.

Our take-home message is that there is a wide range of conditions under which hosts can successfully promote the assembly of a self-sustaining defensive microbiome, which, in turn, supports the general idea that hosts can readily evolve to promote host-beneficial defensive microbiomes.

## Models

We are interested in how the host influences the population dynamics of two different bacterial strains: an antibiotic-producing, antibiotic-resistant beneficial strain (**B**), and a non-producing, sensitive parasitic strain (**P**). (Note that antibiotic producing bacteria must also be antibiotic resistant, or the production would be suicidal.) We model the host implicitly by assuming that it is able to manipulate the composition of its microbiome through resource supply on its surface, upon which colonising individual bacteria compete for space with their neighbours according to their reproduction rates. The host surface further serves as a medium for spatially limited diffusion of the antibiotic. For this, we employ an individual-based model in which we model the host surface as a rectangular grid with toroidal boundary conditions (*N* = M*M) serving as the habitat for colonising bacteria (Fig 1B). Each grid point can be empty or inhabited by a single individual, and interactions take place within the immediate neighbourhood of the focal grid point. Time is measured in units of update steps. We assume that the dynamics of cell reproduction and death processes are much slower than small-molecule dynamics, so the cell populations are updated after *u* (*u*≫1) update steps in antibiotic dynamics, during which the whole grid is updated in all relevant intracellular and extracellular processes related to the small-molecule (antibiotic) dynamics (*N* number of sites). In the cellular update steps, *εN* number of randomly chosen grid cells is updated in the birth and death processes, where *ε* is a small positive number (Fig 1C).

The private resource(s) provided by the host can confer two kinds of benefits to the beneficial strain. We call the first kind (I) **Protected Growth** (mechanisms 1 and 2 from **Introduction***)*, because the parasitic strain is prevented from colonising (certain regions of) the host until time *τ*. Accordingly, in the model, **B** is given preferential access to host-provided space or is solely resistant to host-produced allelochemicals protecting the habitat until time *τ*, after which the host resource is made ‘public’ by also giving the parasitic strain access to the space or by withdrawing the host-produced compounds that have been facilitating **B** and poisoning **P**. We call the second kind of benefit (II) **Enhanced Metabolism** because, in the model, although **P** is allowed to invade from the beginning, **B**’s metabolism is enhanced until time *τ*, after which this enhancement lapses (mechanism 3 from **Introduction**). The simplest outcome of enhanced metabolism is that **B**’s advantage in metabolising host-provided food causes its reproduction rate to be increased by an amount of *r*_B,pr_(*t*) until time *τ*, after which *r*_B,pr_(*t*) = 0 (e.g. *r*_B,pr_(*t*)≥0|*t*<*τ* and *r*_B,pr_(*t*) = 0|*t*≥*τ*), where index pr denotes the private resource. An alternative outcome is that **B** is able to use the host-provided food to increase its own antibiotic-production rate (*ρ*_B_(*t*) = *ρ*_B,pr_(*t*)+*ρ*_B,0_), without incurring higher unit costs. Thus, similar to above, we distinguish a higher production rate fuelled by host-provided resource (*ρ*_B,pr_(*t*)≥0|*t*<*τ*), and a lower, baseline production rate when the resource is not supplied after time *τ* (*ρ*_B,pr_(*t*) = 0|*t*≥*τ*). Naturally, *ρ*_B,0_>0, while the production rate of the non-producing strain is always zero (*ρ*_P_(*t*) = 0). (Alternatively, but not modelled here, the resource could allow the antibiotic to be effective at a lower threshold concentration before *τ* and at higher level after *τ*, which would give similar results to the previous).

### Dynamics of the antibiotic molecules

The beneficial strain produces and exports antibiotic at rate *ρ*_B_, into the extracellular environment, resulting in a distribution of concentrations *A*^Ext^(*i*,*t*) at position *i* at time *t*.

The molecules are taken up by the cells at rates *α*_B_ and *α*_P_ (*α*_B_≤*α*_P_) by the **B** and the **P** strains, respectively, resulting in an *A*^Int^(*i*,*t*) interior concentration within the cell at position *i* at time *t*. The cells decompose the intracellular antibiotics at rates *γ*_B_ and *γ*_P_ (*γ*_B_≤*γ*_P_), and they can also perform active outbound transport, i.e. controlled efflux, to release intracellular antibiotics at rates *β*_B_ and *β*_P_ (*β*_B_≤*β*_P_). The antibiotics decay at rate *φ* in the environment.

The model implements the three major antibiotic-resistance mechanisms: (a) reduced influx through the membrane (*α*_B_), (b) a higher rate of intracellular decomposition and neutralisation (*γ*_B_), and (c) increased efflux of the molecules (*β*_B_), and combinations of these mechanisms [76, 83–86].

We first assume that the antibiotic molecules are point-like particles moving on a host-surface plane. Consequently, we can use reaction-diffusion dynamics to describe change in the extracellular antibiotic concentration *A*^Ext^(**x**,*t*) at points **x** = (*x*,*y*) (representing the coordinates on a surface) and time *t*

$$\frac{\partial A^{Ext}(x,t)}{\partial t}=D\left(\frac{\partial^{2}A^{Ext}(x,t)}{\partial x^{2}}+\frac{\partial^{2}A^{Ext}(x,t)}{\partial y^{2}}\right)+F(A^{Ext}(x,t))$$ (1)

where the first term on the right hand side is the diffusion term, and *F*(*A*^*Ext*^(**x**,*t*)) is the reaction term, which depends on the extracellular antibiotic concentration (*A*^*Ext*^(**x**,*t*)) and the positions and types of the cells. Using the above defined parameters and dynamical processes, we can write

$$F\left(A^{Ext}(x,t)\right)=\sum_{i=1}^{N}\left(\rho_{*}(t)+\beta_{*}A^{Int}(i,t)-\alpha_{*}A^{Ext}(i,t)\right)\delta (x-i)-\varphi A^{Ext}(i,t),$$ (2)

where the antibiotic sources and sinks are summed in the parentheses, *i* is the position of a cell among the *N* cells, which can either be **B** or **P** denoted by * in the bottom index where applicable, *A*^Int^(*i*,*t*) is the intracellular concentration of the antibiotic at position *i*, and *δ* is the Dirac delta [87]. Since in our case the birth and death processes and the spatial positions of particles are given by other complex interaction dynamics, writing down the complete dynamics of the system leads to an analytically intractable model. Therefore, we next implement the time-and-space-discretised dynamics of antibiotic concentration at site *i* on the rectangular grid and at time *t*+Δ*t* in the extracellular environment as

$$\begin{array}{c} A^{Ext}(i,t+\Delta t)=A^{Ext}(i,t)\\ +\left[\frac{D}{\Delta x^{2}}(\sum_{j=1}^{v}A^{Ext}(j,t)-{vA}^{Ext}(i,t))+(\rho_{*}(t)+\beta_{*}A^{Int}(i,t)-\alpha_{*}A^{Ext}(i,t)-\varphi A^{Ext}(i,t))\theta (i)\right]\Delta t \end{array}$$ (3)

where the first term corresponds to the diffusion of antibiotics according to the discretised diffusion algorithm between the four nearest neighbouring points (*v* = 4) (Neumann-neighbourhood: north, south, east, west); Δ*x* is the spatial resolution, and Δ*t* is the time resolution. The diffusion rate of the antibiotics, *D*, is measured in the unit of *x*^2^/*t*, where *x* denotes the spatial resolution, here one cell of the grid, and *t* stands for time measured as an update step. *θ*(i) takes the value one if there is a cell at the site *i*, else being zero. The dynamics of intracellular concentration of the antibiotic at the site *i* can be written as

$$A^{Int}(i,t+\Delta t)=A^{Int}(i,t)+\left(\alpha_{*}A^{Ext}(i,t)-\beta_{*}A^{Int}(i,t)-\gamma_{*}A^{Int}(i,t)\right)\Delta t.$$ (4)

Naturally *A*^Int^(*i*,*t*+Δ*t*) = *A*^Int^(*i*,*t*) = *A*^*Ext*^(*i*,*t*) = 0 if there is no cell at site *i*.

### Growth dynamics of the cells

For the birth and death processes, we define the reproduction or growth rate of the antibiotic-producing (**B**) and non-producing (**P**) strains respectively as

$$\begin{array}{c} r_{B}(i,t)=r_{B,0}+r_{B,pr}(t)-c,\\ r_{P}(i,t)=r_{P,0}-\lambda (a,T,k,A^{Int}(i,t)) \end{array}$$ (5)

where *c* is the decrease in reproduction rate because of the costly processes of antibiotic production and resistance. The reproduction rates *r*_B,0_, *r*_P,0_, and *r*_B,pr_(*t*) correspond to normal (baseline) and temporarily increased resource conditions, respectively. We assume *r*_B,0_ = *r*_P,0_; different assumptions would only rescale the value of *c* (see S5 Fig in the **Supplementary Information** for different choices of *r*_B,0_). The effect of the antibiotic *λ*(*a*,*T*,*k*,*A*^Int^(*i*,*t*)) on the **P** strain’s reproduction rate depends on the critical threshold (*T*), the maximum effect (*a*), the steepness of the dosage effect (*k*), and the actual intracellular concentration of the antibiotic in the sensitive cell at the site *i* (*A*^Int^(*i*,*t*)). Following empirical observations [61], we define a general sigmoid function for the effect of the antibiotic:

$$\lambda \left(a,T,k,A^{Int}(i,t)\right)=a/\left[1+exp(-k(A^{Int}(i,t)-T))\right]$$ (6)

### Dynamics of the population

Population dynamics are represented by a death-birth process in which a randomly chosen focal individual at site *i* dies, and individuals from its Moore neighbourhood (8 nearest neighbours, *w* = 8) can reproduce and place a progeny into this focal empty site, with probability proportional to their reproduction rates

$$p(i)=r_{\xi }(i,t)/\sum_{j=1}^{w}r_{\xi }(j,t),\;\mathrm{where}\;(\xi \epsilon \{P,B\})$$ (7)

At the beginning of the simulation, the beneficial strain is represented in low numbers (*n*_B,0_), and the parasitic strain is missing (*n*_P,0_ = 0).

### Invasion tests

We carried out two sets of invasion tests to demonstrate how host-provided private resources can result in self-sustaining, beneficial microbiomes, even if the private resource itself eventually diminishes. In the first test, we used time, while in the second, we used colony size as the signal to switch from private to public resources, or in other words, to stop the host’s selective support for the beneficial strain.

#### Invasion test 1. Time-limited supply of private resources

We model the two kinds of benefits conveyed by the private resources, as discussed earlier, the (I) Protected Growth of the beneficial strain for *τ* time and (II) the Enhanced Metabolism of the beneficial strain for *τ* time, either leading to (IIa) a higher population growth rate by the beneficial strain or to (IIb) an increased antibiotic production by the beneficial strain.

In the **Protected Growth** scenario, before *τ*, an *s*_+_ proportion of cohesive space on the host surface (*s*_+_ = *ss*/*N*, where *ss* is the number of protected sites) provides a safe growth opportunity for the beneficial strain, as individuals from the parasitic strain are prevented from invading (strict and pseudo-vertical transmission), or parasitic individuals invading this region get killed off (via host-provided allelochemicals). However, after *τ* time has passed, the parasitic strain is finally allowed to gain a foothold on the grid. In other words, the private space resource is made public at time *τ*. During an invasion attempt, we place *n*_P,t_ number of individuals around a randomly selected focal grid point in a connected cluster with probability *f* in each time step (if there are empty places, subsequent individuals will be placed next to the focal site, but non-empty grid points can also be occupied if no empty place is available). In **the Enhanced Metabolism** scenario, the beneficial strain experiences increasing advantages of *r*_+_ = (*r*_B,pr_(*t*)+*r*_B,0_)/*r*_B,0_ or *ρ*_+_ = (*ρ*_B,pr_(*t*)+*ρ*_B,0_)/*ρ*_B,0_ for *τ* time, respectively, and *n*_P,t_ number of parasitic-strain individuals are allowed to invade with probability *f* in each time step, starting from the beginning (*κ* = 1).

#### Invasion test 2. Protected growth of the beneficial strain to a minimum colony size

Here, we let the host resource, the habitat, be private until the beneficial strain reaches a minimum colony size, which we call the Colony Size at Invasion (*CSI*). We only allow the parasitic strain to start invading empty places after the resident **B** strain’s colony size has grown to the *CSI* (*CSI* = *q*/*N*, where *q* is the number of sites inhabited by **B**). The invasion proceeds with probability *f* starting from *κ* and with *n*_P,t_ number of invaders until the grid is fully occupied by individuals. As a motivating example, one can think of a small host ‘crypt’ or ‘symbiont capsule’ [43–45] in which the beneficial strain is initially housed. The strain eventually outgrows the crypt, or the new-born host opens up the capsule, releasing the symbionts, which then colonise the host surface [43–45]; at this point, the host can only provide resources in a way that makes them publicly available. The capsule serves *de facto* as a private resource, as the symbionts are, through specialized mechanisms, encapsulated from the progenitor’s symbiont community, and these capsules are only broken upon the inhabitation of the new habitat [43–45], which is now available to all bacterial strains.

## Results

The colony growth follows a logistic growth dynamic in the model (Fig 1C). Depending on the choice of *ε*, we observe full colonisation of the surface within a given timeframe. Without involving the effects of the host-provided private resources for the beneficial strain, and without the reproduction-rate reducing effect of the antibiotic on the parasitic strain, the faster growing type would quickly become dominant in the habitat, which, in our case, is the parasite, as it pays no cost of producing any compounds. To better investigate the competition dynamics between the two types on a fine timescale, we choose *ε* = 0.01 for our further investigations.

### Invasion test 1. Time-limited supply of private resources

**Fig 2:**
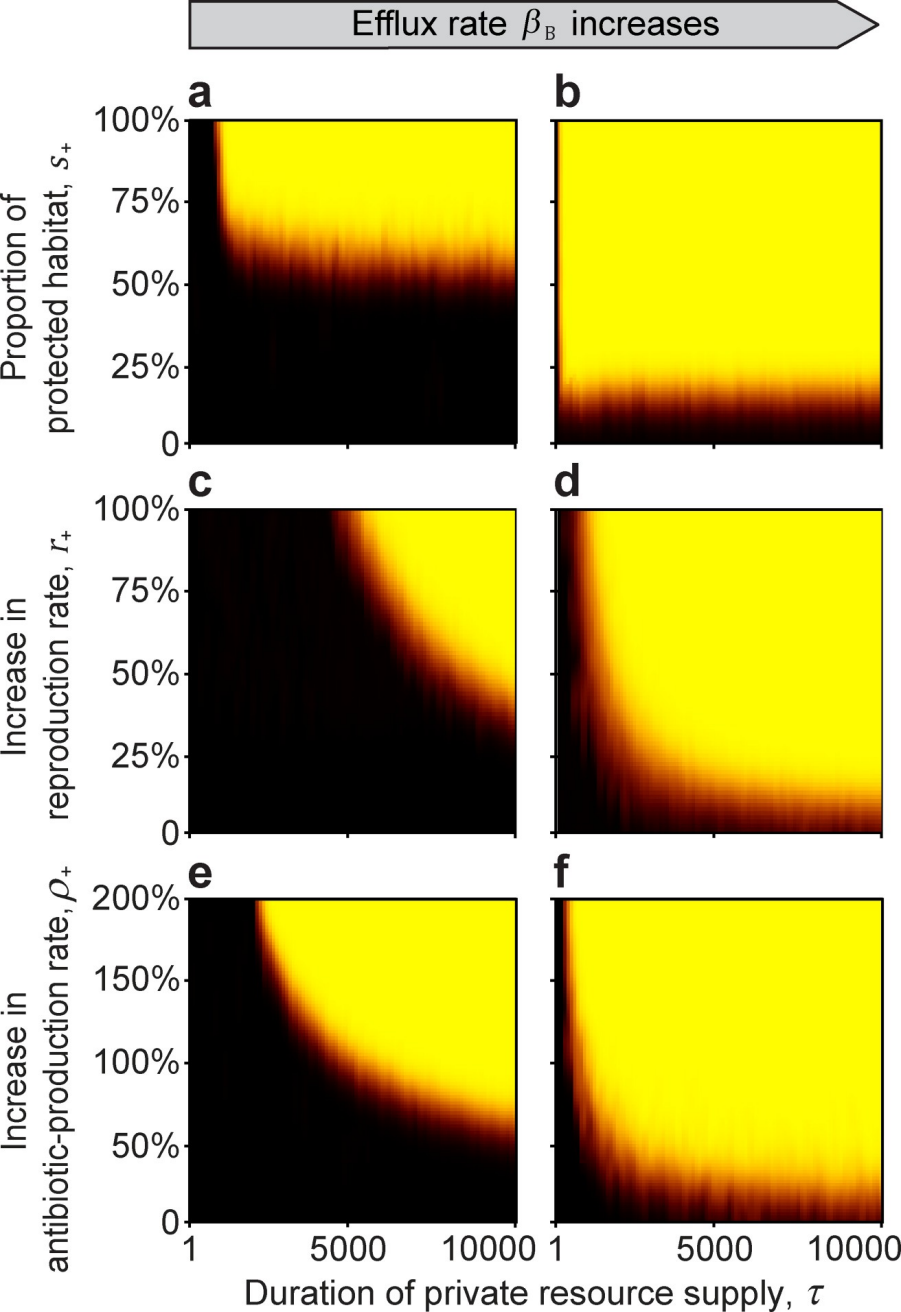
The effect of a private resource supplied by the host for a limited time *τ* (Invasion test 1). Black areas indicate parameter space where the non-producing parasitic strain can invade, and the yellow shading indicates that the beneficial strain is able to resist invasion. Orange to red colours indicate mixed outcomes. In general, the beneficial strain dominates over a larger proportion of the parameter space as the duration of the private resource supply lengthens, regardless of whether the beneficial strain enjoys outright protected growth (**a**, **b**), an increased rate of population growth (**c**, **d**), or an increased rate of antibiotic production (**e**, **f**). The efflux of accumulated intracellular antibiotic in the antibiotic-producing beneficial strain also aids beneficial-strain dominance (*β*_B_ = 0 for **a**, **c**, **e**, and *β*_B_ = 0.25 for **b**, **d**, **f**). Simulations were run with 5 replicates for 100 000 generations or until the population was homogenous. Model parameters are: *r*_B,0_ = 0.8, *r*_P,0_ = 0.8, *c* = 0.1, *ρ*_B,0_ = 1, *α*_B_ = 0.5, *α*_P_ = 0.5, *β*_P_ = 0, *γ*_B_ = 0.4, *γ*_P_ = 0.4, *φ* = 0.3, *D* = 5, *a* = 1, *T* = 1, *k* = 25, *N* = 10 000, *n*_B,0_ = 100, *n*_P,t_ = 10, *κ* = 1 *f* = 0.01, Δ*t* = 1/10, *u* = 100, *ε* = 0.01, and *r*_+_ = 0, *s*_+_ = 0, *ρ*_+_ = 0 when applicable.

As discussed in the **Introduction**, the host has multiple mechanisms by which it can provide private resources. We find that protecting initial growth (Fig 2A and 2B), increasing the reproduction rate (Fig 2C and 2D), and/or enhancing the antibiotic effectiveness (Fig 2E and 2F) of the beneficial strain, can all result in a self-sustaining, beneficial-strain-dominated microbiome that is resistant to invasion even after the host resource is made public (at time *τ*) and the beneficial strain starts to experience a competitive disadvantage due to its costs of antibiotic production and of expressing its antibiotic-resistance traits. In all three scenarios, the longer the time *τ* that the resource is private (Fig 2, x-axis), the less of an advantage, in the form of protected growth (*s*_+_), increased population growth (*r*_+_), or increased antibiotic production (*ρ*_+_) (Fig 2, y-axis), is required for the beneficial strain to be able to resist invasion after the resource becomes public. This is because invasion resistance increases with the size of the beneficial colony and with the concentration of antibiotic that the colony produces and transports into the environment.

We also observe that if the physiological mechanism of resistance by the beneficial strain to its own antibiotic is efflux, this can additionally enhance invasion resistance, even if the supply time is short and the advantage conferred by the private resource is small (Fig 2A, 2C, 2E vs. 2B, 2D and 2F). The reason is that re-exporting any ingested antibiotic increases the environmental concentration of antibiotic, which aids suppression of invading parasitic strains.

### Invasion test 2. Protected growth of the beneficial strain until a minimum colony size

**Fig 3:**
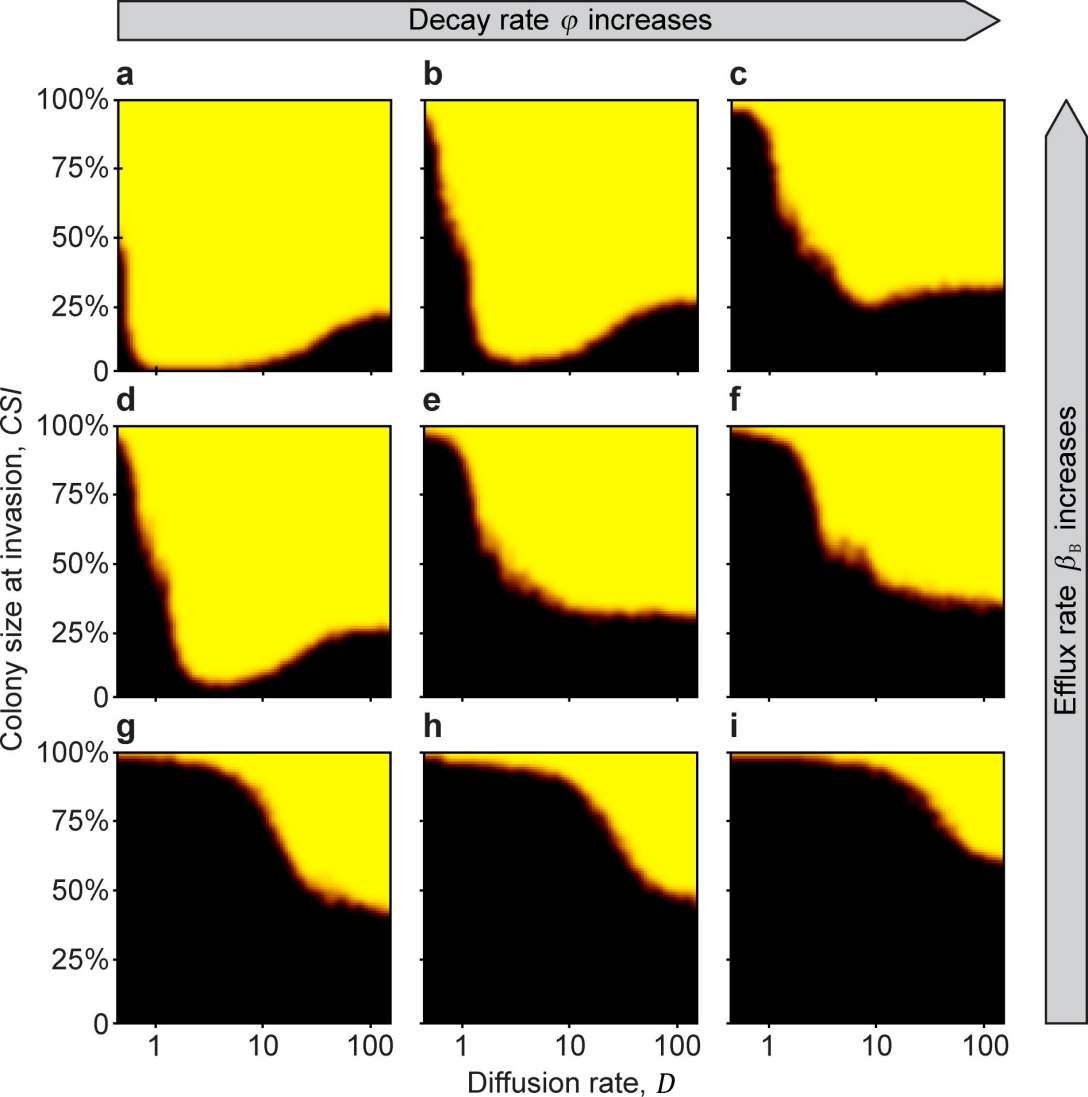
The Minimal Sustainable Colony size (*MSC*) (Invasion test 2). Invasion is initiated when the beneficial-strain colony reaches a defined size (*CSI*) and continues until the habitat is fully colonized by either the beneficial or the parasitic strains. The *MSC* is represented by the orange-red border separating the yellow (**B** wins) and black (**P** wins) regions. From left to right (**a**→**c**, **d**→**f**, and **g**→**i**), the extracellular decay rate of the antibiotic *φ* increases (*φ* = 0.2,0.25,0.3). From top to bottom (**a**→**g**, **b**→**h**, and **c**→**i**), the efflux rate *β*_B_ decreases (*β*_B_ = 1,0.5,0). Simulations were run with 3 replicates for 100 000 generations, or until the population was homogenous. Black areas indicate parameter space where the parasitic strain can invade, yellow indicates parameter space where the antibiotic-producing beneficial strain successfully resists invasion, and orange areas correspond to mixed outcomes. Model parameters are: *r*_B,0_ = 0.8, *r*_P,0_ = 0.8, *c* = 0.4, *ρ*_B,0_ = 1, *α*_B_ = 0.5, *α*_P_ = 0.5, *β*_P_ = 0, *γ*_B_ = 0.4, *γ*_P_ = 0.4, *D* = 5, *a* = 1, *T* = 1, *k* = 25, *N* = 10 000, *n*_B,0_ = 100, *n*_P,t_ = 10, *κ* = 1, *f* = 0.01, Δ*t* = 1/10, *u* = 100, *ε* = 0.01, *r*_+_ = 0, *s*_+_ = 0, *ρ*_+_ = 0, and *τ* = 0.

**Fig 4:**
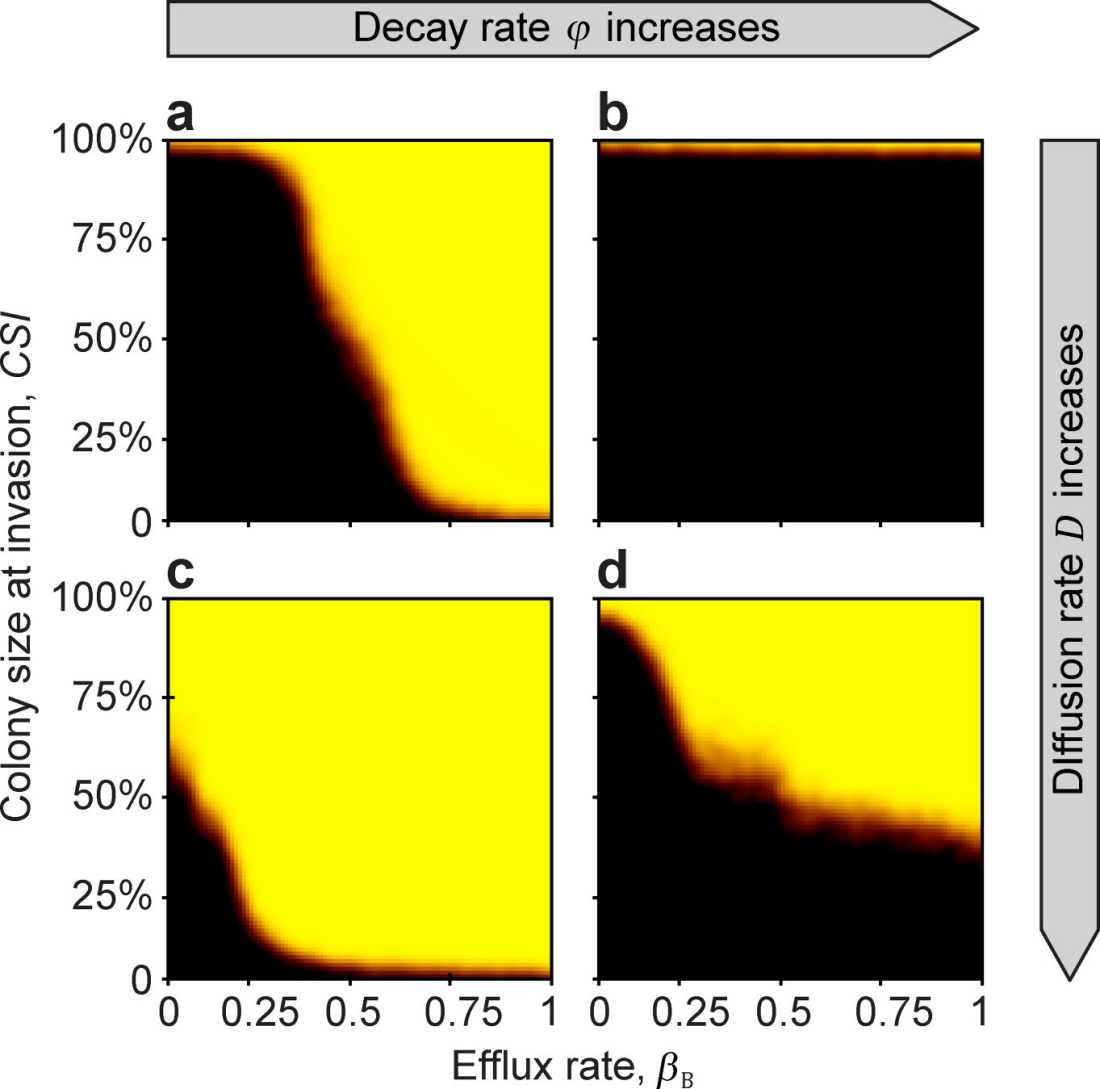
The effect of efflux rate, decay rate, and diffusion rate on the *MSC*. At low diffusion rates (upper row), efflux rate limits the success, while at large diffusion rate (bottom row), colony size is the more limiting factor. From left to right (**a**→**b**, **c**→**d**) extracellular decay rate increases (*φ* = 0.7 and 0.9). From the top to the bottom (**a**→**c**, and **b**→**d**), diffusion rate increases (*D* = 0.5 and 12), respectively. Model parameters are: *r*_B,0_ = 0.8, *r*_P,0_ = 0.8, *c* = 0.1, *ρ*_B,0_ = 1, *α*_B_ = 0.6, *α*_P_ = 0.6, *β*_P_ = 0, *γ*_B_ = 0.3, *γ*_P_ = 0.3, *a* = 1, *T* = 1, *k* = 25, *N* = 10 000, *n*_B,0_ = 100, *n*_P,t_ = 10, *κ* = 300, *f* = 0.01, Δ*t* = 1/10, *u* = 100, *ε* = 0.01, *r*_+_ = 0, *s*_+_ = 0, *ρ*_+_ = 0, and *τ* = 0.

**Fig 5:**
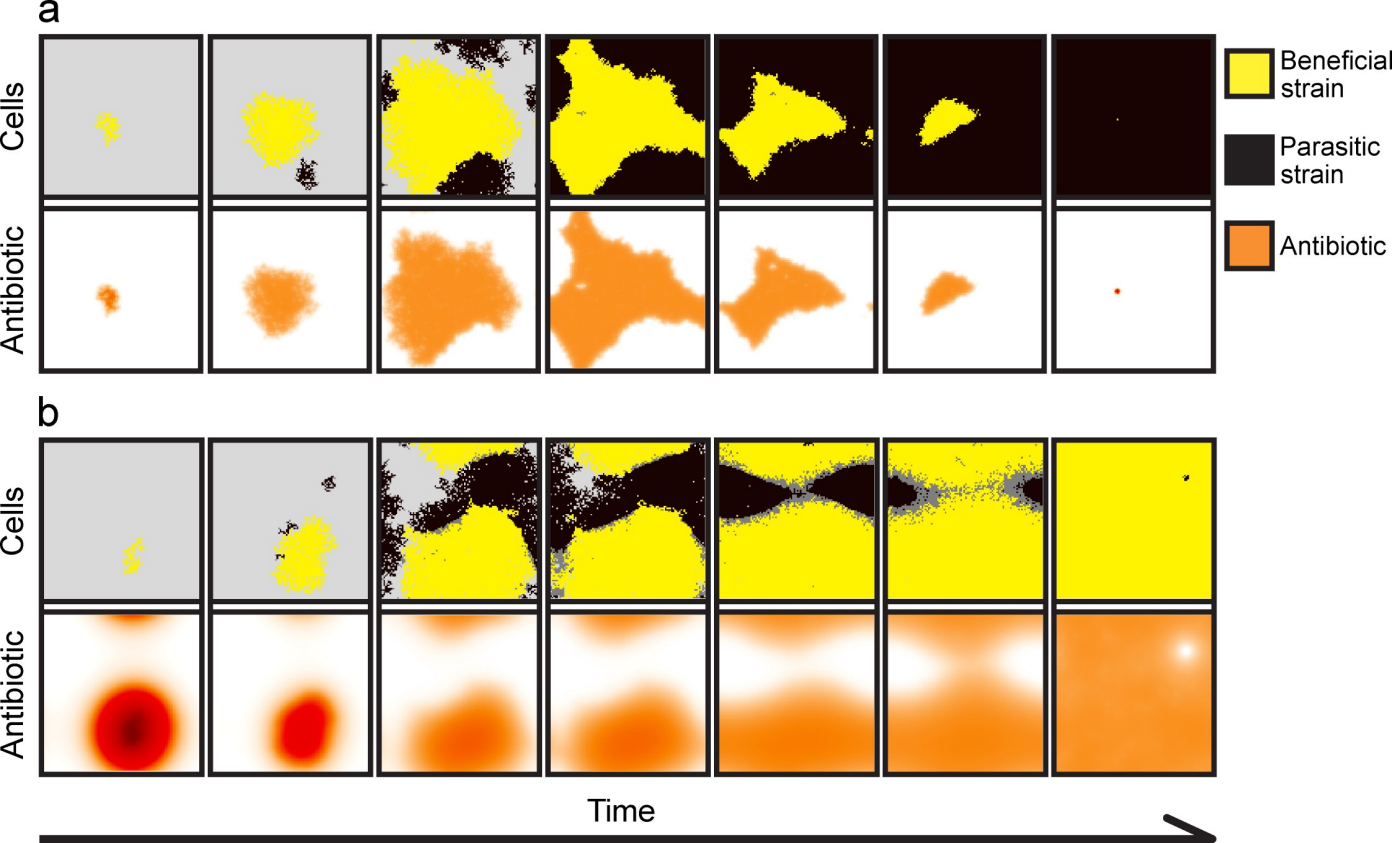
**Spatial dynamics for (a) low and (b) high diffusion rates.** (**a**) A low diffusion rate (*D* = 0.5) reduces the protective effect of the antibiotic (orange shading, lower panels), and the parasitic strain (black shading, upper panels) can invade the beneficial strain (yellow shading, upper panels). (**b**) A high diffusion rate (*D* = 50) allows the beneficial strain to resist invasion, as considerable amount of antibiotic (lower panels) diffuses beyond the colony boundaries. Antibiotic concentration ranges between zero (white), to intermediate (red-orange), to maximal concentrations (brown-black). Poisoned (cells with *r*_P_<0.05) but not yet removed parasitic cells are coloured dark grey. In these simulations, the beneficial colony was allowed *κ* = 300 time steps to grow before invasion. The snapshots of the simulations are taken every 500 update steps. Model parameters are: *r*_B,0_ = 0.8, *r*_P,0_ = 0.8, *c* = 0.4, *ρ*_B,0_ = 1, *α*_B_ = 0.5, *α*_P_ = 0.5, *β*_B_ = 0.4, *β*_P_ = 0, *γ*_B_ = 0.4, *γ*_P_ = 0.4, *φ* = 0.25, *a* = 1, *T* = 1, *k* = 25, *N* = 10 000, *n*_B,0_ = 100, *n*_P,t_ = 10, *κ* = 300, *f* = 0.01, Δ*t* = 1/10, *u* = 100, *ε* = 0.01, *r*_+_ = 0, *s*_+_ = 0, *ρ*_+_ = 0, and *τ* = 0.

Consistent with the results from Invasion test 1, if the beneficial colony successfully reaches a critical size (the Minimum Sustainable Colony size: *MSC*), it becomes resistant to invasion over a wide range of parameters after the private resource is made public (Fig 3). Again, having antibiotic efflux as the resistance mechanism promotes invasion resistance (Figs 3 and 4), whereas (and intuitively) a higher rate of extracellular decay of antibiotic counteracts this effect (Figs 3 and 4). When a large amount of antibiotic is in the environment, because efflux is high and decay is low (Fig 3A and Fig 4A and 4C), the beneficial strain is able to dominate over a wide range of diffusion rates. However, when the extracellular-decay rate is high, only high diffusion rates allow the beneficial strain to dominate (Fig 4D). This is because at low diffusion rates, the antibiotic produced in the centre of the colony is lost due to decomposition before it diffuses to the colony edge, where it would have attacked invaders. In contrast, at high diffusion rates, more of the antibiotic produced by cells deeper in the colony reaches the invasion front at the edge (Figs 3, 4, and 5).

The complement to this result is that if the diffusion rate is low, then even a large colony size does not necessarily guarantee success unless the efflux rate is also high enough (Fig 4A and 4B). Essentially, if antibiotic efflux is used as the resistance mechanism by the beneficial cells, this can substitute for outright diffusion of the antibiotic, allowing the antibiotic to reach the colony edge, where it can suppress invaders (Fig 4).

### Non-monotonous effect of diffusion

Interestingly, under some conditions there is a non-monotonous effect of diffusion rate on invasion resistance, such that the Minimum Colony Size (*MSC*) can be much smaller for medium-level diffusion rates. For example, looking at Fig 3B, for low antibiotic diffusion rates (values 0−1 on the x-axis), the *MSC* is close to 100%; that is, the colony can resist invasion only if more than 95% of the available habitat is already occupied by the producers; otherwise, parasites displace the whole population of antibiotic producers. Similarly, for high diffusion rates (values *D* = 80−100 on the x-axis), although smaller, a considerable colony size still has to be reached. However, the *MSC* curve reaches a minimum between low and high diffusion rates, such that only a 1−10% *MSC* is enough to resist invasion (Fig 3B).

The important result is that for any intermediate efflux and decay parameters coupled with intermediate diffusion rates, colonies with practically any non-zero initial size can withstand parasite invasion (Fig 3A, 3B and 3D). This nonlinearity occurs because, in general, diffusion carries antibiotic to the edge of the antibiotic-producing colony, where it can act against invading **P** strains, but diffusion also carries antibiotic away from the edge of the colony. An intermediate diffusion rate turns out to maximise the amount of antibiotic at the fighting front (see S1–S6 Figs in the **Supplementary Information** for further results of different parameter combinations).

## Discussion

The composition of host-associated microbiomes has been shown to correlate with host health status and fitness [4, 88–94], and thus, there is likely to be strong selection on host species to evolve mechanisms that favour the assembly of certain kinds of microbiomes over others [11, 12, 27]. Here we have explored how a host can favour the assembly of a defensive microbiome that is persistently dominated by antibiotic-producing bacteria [7, 23, 77, 95].

We argue that a host can take advantage of an ecological phenomenon known as *‘*community bistability’: when two species compete via interference, such as when a bacterial species uses antibiotics to hinder a competitor, the winner depends partially on the initial population sizes of the two competitors [9]. If the antibiotic-producer initially establishes a larger population in the new habitat, it can collectively produce a sufficient amount of antibiotic to suppress its competitor and grow until the space of opportunity vanishes for the non-producer. In contrast, if the non-producer species starts with the larger or competitively superior population, then the small amount of antibiotic produced by a small colony of a producer is insufficient to suppress the non-producer, and the non-producer wins. It follows that by using an antibiotic-producer as the initial (or ‘priming’) strain of the microbiome, a host can narrow down the variety of strains able to invade this already established environment [4, 5, 9, 11]. The host is thus efficiently able to canalise the composition of the emerging microbiome. Such priming effects have been demonstrated in various experimental systems [25, 37, 39, 96]. Our argument, in a nutshell, is that an effective way for hosts to guide microbiome assembly is by manipulating initial conditions, resulting in a cascade of bacterial community dynamics that ultimately favour some kinds of microbiomes over others, which will, in turn, affect host fitness. Another way of thinking about this is through the lens of game theory [9, 13]. The host is able to ‘screen-in’ antibiotic-producing bacteria by exploiting a fundamental correlation: bacterial strains that make lots of antibiotics are both superior interference competitors against other bacteria and also likely to produce compounds that are useful for host defence.

In this study, we have integrated local interactions and the explicit spatial dynamics of cellular and chemical components with the original phenomenological model that laid the foundations of the theory [9]. In this now more realistic model, even for large populations, the number of directly interacting cells is relatively modest, and thus, the spatial correlations of active agents determine dynamics meaningfully [5, 78]. Furthermore, such an integrated, spatially-explicit model allows us to understand the effect of different antibiotic-resistance mechanisms [75, 83–86, 97] on the microbiome assembly, and to investigate how attributes of the host surface, which govern the diffusion dynamic of the antibiotic, can modify the outcome. We have also widened the applicability of Scheuring and Yu’s original model [9] by reviewing multiple mechanisms allowing a host to prime a defensive microbiome, even if the beneficial strain can only be recruited from the environment (horizontal transmission); the original model made the restrictive assumption that the beneficial strain is strictly vertically transmitted.

We have corroborated the earlier results [9, 13] that antibiotic producers and non-producers can form a bistable system and that the outcome of competition depends on their reproduction rates, how effectively the host is able to selectively promote the beneficial strain, and the initial ratio of the two strains [9]. Once the antibiotic producer is able to gain dominance, in such a system it can remain dominant for a lifetime, even if the host-provided private resource vanishes or becomes public. The current model also shows that localized interactions, which is an important realism that had been ignored in the simpler model [9], do not impede this dominance because the antibiotic itself can diffuse to the colony edge to inhibit invaders. This effect is strengthened when the mode of resistance employed by the producers is antibiotic efflux.

We also show with the current model that the host resource only needs to remain private for a finite critical time, basically until the beneficial colony reaches a Minimal Sustainable Colony Size (*MSC*), at which point it becomes resistant to a given rate of invasion. The critical time and/or the *MSC* depends on the physiochemical properties of the system, most importantly the decomposition, decay, diffusion, and efflux rates of the antibiotic, and the advantage provided to the beneficial colony by the private resource, all deriving from the fact that colony size determines the amount of antibiotic produced.

Our brief review of the literature suggests that multiple forms of ‘private resource’ exist, including food, space, and host-provided compounds that harm undesired strains. Nonetheless, privacy of resources is inherently difficult and costly to achieve, and it is therefore realistic to assume that any host-provided resources will eventually become public. This inevitable transition from private to public, which intuitively might be expected to allow the successful invasion and establishment of parasitic strains, does not in fact do so, because of bistability. After a beneficial colony establishes itself, a public resource is in practice only enjoyed by the winner, the beneficial colony.

Finally, we show that an intermediate diffusion rate can maximise the amount of antibiotic accumulating at the colony edge. Our findings suggest that the attributes of the host surface, for example the diffusion rate, can either increase or reduce the effect range of the antibiotic [98]. As there is no conflict of interest between antibiotic-producer and host, their coevolution is expected to optimise the diffusion speed, and hence the effectiveness, of the antibiotic. Overall, evolutionary optimisation can act by minimising the host investment required to attain a beneficial microbiome, by reducing the duration of a private resource supply, and by evolving the optimal physiochemical properties of the habitat, the host surface. If so, then we might also expect that the co-evolution of host and preferred strains results in an efficient and well-conducted build-up of a beneficial microbiome, an orchestrated symbiosis that efficiently narrows down the enormous number of possible scenarios to canalise the emergence of a microbiome towards the most favourable one.

## Supporting information

S1 Fig The effect of different decay rates (*φ*) on the effectiveness of the private resource for the beneficial strain in the form of increased antibiotic-production rate for *τ* time. High extracellular decay rates reduce the effectiveness of the antibiotic and thus decrease the effectiveness of the help provided by the host to the beneficial microbe for *τ* time. Therefore, to compensate for higher decay rates, higher effort is needed from the host either in the form of more help (along the y-axis) or in the form of same amount of help provided for longer time (x-axis). Results are shown for (**a**) zero (*β*_B_ = 0) and for (**b**) modest (*β*_B_ = 0.25) efflux rates. The upper right areas correspond to beneficial-dominated outcomes, while the bottom left corners correspond to parasite-dominated outcomes. The light (ochre) shadings represent the regions in which the beneficial strain wins for more than 50% of the simulations, and the darker (black) shaded areas mark the parameter combinations in which the parasitic strain wins for the majority of simulations. The black lines mark the boundaries separating these two regions. Model parameters are: *r*_B,0_ = 0.8, *r*_P,0_ = 0.8, *c* = 0.1, *ρ*_*B*,0_ = 1, *α*_B_ = 0.5, *α*_P_ = 0.5, *β*_P_ = 0, *γ*_B_ = 0.4, *γ*_P_ = 0.4, *D* = 5, *a* = 1, *T* = 1, *k* = 25, *N* = 10 000, *n*_B,0_ = 100, *n*_P,t_ = 10, *κ* = 1, *f* = 0.01, Δ*t* = 1/10, *u* = 100, *r*_+_ = 0, and *s*_+_ = 0.
(TIF)

S2 Fig The trade-off between the extracellular decay rate (*φ*) and the intracellular decomposition rate (*γ*). High decay rates reduce the effect of the antibiotic; hence, the antibiotic is effective against the parasitic strain only if the decomposition rate is low and the antibiotic accumulates more quickly in the sensitive cells to lethal concentrations. Results are shown for (**a**) low (*D* = 0.5) and (**b**) modest (*D* = 5) diffusion rates. The upper right areas correspond to parasite-dominated outcomes, and the bottom left areas correspond to beneficial-dominated outcomes. The light (ochre) shadings represent the regions in which the beneficial strain wins for more than 50% of the simulations, and the darker (black) shaded areas mark the parameter combinations in which the parasitic strain wins for the majority of simulations. The black lines mark the boundaries separating these two regions. Model parameters are: *r*_B,0_ = 0.8, *r*_P,0_ = 0.8, *c* = 0.1, *ρ*_*B*,0_ = 1, *α*_B_ = 0.5, *α*_P_ = 0.5, *β*_P_ = 0, *a* = 1, *T* = 1, *k* = 25, *N* = 10 000, *n*_B,0_ = 100, *n*_P,t_ = 10, *κ* = 1, *f* = 0.01, Δ*t* = 1/10, *u* = 100, *τ* = 0, *r*_+_ = 0, *s*_+_ = 0, and *ρ*_+_ = 0.
(TIF)

S3 Fig The trade-off between the cost of antibiotic production (*c*) and the effort provided by the host to aid the beneficial strain (*r*_+_). The higher the cost of producing the antibiotic (x-axis), the more support from the host, in the form of private resource increasing the growth rate of the beneficial (y-axis) is required to secure the dominance of the beneficial strain. The longer that the private resource is provided (***τ*)** the higher the cost that can be tolerated. Results are shown for (**a**) low (*β*_B_ = 0), (**b**) modest (*β*_B_ = 0.25), and (**c**) medium (*β*_B_ = 0.5) efflux rates. The upper left areas correspond to beneficial-dominated outcomes, while the right-hand and bottom right areas correspond to parasite-dominated outcomes. The light (ochre) shadings represent the regions in which the beneficial strain wins for more than 50% of the simulations, and the darker (black) shaded areas mark the parameter combinations in which the parasitic strain wins for the majority of simulations. The black lines mark the boundaries separating these two regions. Model parameters are: *r*_B,0_ = 0.8, *r*_P,0_ = 0.8, *ρ*_*B*,0_ = 1, *α*_B_ = 0.5, *α*_P_ = 0.5, *β*_P_ = 0, *γ*_B_ = 0.4, *γ*_P_ = 0.4, *φ* = 0.3, *D* = 5, *a* = 1, *T* = 1, *k* = 25, *N* = 10 000, *n*_B,0_ = 100, *n*_P,t_ = 10, *κ* = 1, *f* = 0.01, Δ*t* = 1/10, *u* = 100, *s*_+_ = 0, and *ρ*_+_ = 0.
(TIF)

S4 Fig The relationship between the increasing cost of antibiotic production (*c*) and the reproduction−rate reducing effect of the antibiotic on the parasitic strain (dosage effect, *a*). The higher the cost of producing the antibiotic (x-axis), the more effective the antibiotic (y-axis) must be to secure the dominance of the beneficial strain (where effectiveness is measured as reducing the growth rate of the parasitic strain). Also, the higher the efflux rate (*β***),** the larger the cost can be tolerated. Very low (**a**; *D* = 0.5) and very high (**c**; *D* = 50) diffusion rates hinder the effectiveness of the antibiotic, hence the highest costs are tolerated at medium (**b**; *D* = 5) diffusion rates. The upper left corners correspond to beneficial-dominated outcomes, while the right-hand and bottom right areas correspond to parasite-dominated outcomes. The light (ochre) shadings represent the regions in which the beneficial strain wins for more than 50% of the simulations, and the darker (black) shaded areas mark the parameter combinations in which the parasitic strain wins for the majority of simulations. The black lines mark the boundaries separating these two regions. Model parameters are: *r*_B,0_ = 0.8, *r*_P,0_ = 0.8, *ρ*_*B*,0_ = 1, *α*_B_ = 0.5, *α*_P_ = 0.5, *β*_P_ = 0, *γ*_B_ = 0.4, *γ*_P_ = 0.4, *φ* = 0.3, *T* = 1, *k* = 25, *N* = 10 000, *n*_B,0_ = 100, *n*_P,t_ = 10, *κ* = 1, *f* = 0.01, Δ*t* = 1/10, *u* = 100, *τ* = 2000, *r*_+_ = 0.4, *s*_+_ = 0, and *ρ*_+_ = 0.
(TIF)

S5 Fig The trade-off between the growth rate of the beneficial strain (*r*_B,0_) and the cost of producing the antibiotic by the beneficial strain (*c*). High reproduction rates allow for higher costs, as the competitive disadvantage caused by the costly antibiotic production can be compensated by higher reproduction rates compared to that of the parasitic strain’s (*r*_P,0_ = 0.8). Higher efflux rates (*β*_B_ = 0 → *β*_B_ = 1) can also help to compensate for a competitive disadvantage caused by lower reproduction rates and costly antibiotic production. Results are shown for (**a**) low (*D* = 0.5), (**b**) medium (*D* = 5), and (**c**) high (*D* = 50) diffusion rates. Increasing the diffusion rate further improves the effectiveness of the antibiotic, and hence the beneficial can dominate even with relatively high cost and low reproduction rates, until a certain point. Very high diffusion rates, on the other hand, hinder the effectiveness of the antibiotic. The upper left areas correspond to parasite-dominated outcomes, while the bottom right corners correspond to beneficial-dominated outcomes. The light (ochre) shadings represent the regions in which the beneficial strain wins for more than 50% of the simulations, and the darker (black) shaded areas mark the parameter combinations in which the parasitic strain wins for the majority of simulations. The black lines mark the boundaries separating these two regions. Model parameters are: *r*_P,0_ = 0.8, *c* = 0.1, *ρ*_*B*,0_ = 1, *α*_B_ = 0.5, *α*_P_ = 0.5, *β*_P_ = 0, *γ*_B_ = 0.4, *γ*_P_ = 0.4, *φ* = 0.3, *D* = 5, *a* = 1, *T* = 1, *k* = 25, *N* = 10 000, *n*_B,0_ = 100, *n*_P,t_ = 10, *κ* = 1, *f* = 0.01, Δ*t* = 1/10, *u* = 100, *τ* = 2000, *r*_+_ = 0.4, *s*_+_ = 0, and *ρ*_+_ = 0.
(TIF)

S6 Fig The relationship between invasion probability per update (*f*) and the start of invasion of the parasitic strain (*τ*). During these simulations, the invasion of the parasitic strain is blocked on the entire host surface, thus directing the host-provided resource entirely to the beneficial strain, until time *τ* (*τ* = *κ*). The higher the probability of invasion by the parasite (y-axis), the more time is necessary for the beneficial to reach a colony size big enough to resist invasion (x-axis). The higher the extracellular decay rate is (*φ*), the more time is required for the beneficial colony to become resistant to invasion, but this disadvantage can be compensated by high efflux rates (*β*_B_ = 0 for **a** and *β*_B_ = 0.25 for **b**). There is a limit, however, to how much invasion pressure the system can withstand; as can be seen, for high decay rates and above a certain invasion pressure (high *f* values along the y-axis), even long-lasting host support cannot result in dominance of the beneficial strain. The upper left corners correspond to parasite-dominated outcomes, while the bottom right areas correspond to beneficial-dominated outcomes. The light (ochre) shadings represent the regions in which the beneficial strain wins for more than 50% of the simulations, and the darker (black) shaded areas mark the parameter combinations in which the parasitic strain wins for the majority of simulations. The black lines mark the boundaries separating these two regions. Model parameters are: *r*_B,0_ = 0.8, *r*_P,0_ = 0.8, *c* = 0.1, *ρ*_*B*,0_ = 1, *α*_B_ = 0.5, *α*_P_ = 0.5, *β*_P_ = 0, *γ*_B_ = 0.4, *γ*_P_ = 0.4, *D* = 5, *a* = 1, *T* = 1, *k* = 25, *N* = 10 000, *n*_B,0_ = 100, *n*_P,t_ = 10, Δ*t* = 1/10, *u* = 100, *r*_+_ = 0, *s*_+_ = 1, and *ρ*_+_ = 0.
(TIF)

## References

[1] Costello EK, Stagaman K, Dethlefsen L, Bohannan BJM, Relman DA. The application of ecological theory toward an understanding of the human microbiome. Science. 2012;336: 1255–1262. 10.1126/science.1224203 22674335 PMC4208626

[2] Levy R, Borenstein E. Metabolic modeling of species interaction in the human microbiome elucidates community-level assembly rules. Proc Natl Acad Sci U S A. 2013;110: 12804–12809. 10.1073/pnas.1300926110 23858463 PMC3732988

[3] Weber MF, Poxleitner G, Hebisch E, Frey E, Opitz M. Chemical warfare and survival strategies in bacterial range expansions. J R Soc Interface. 2014;11: 20140172 10.1098/rsif.2014.0172 24806706 PMC4032534

[4] McNally L, Brown SP. Building the microbiome in health and disease: niche construction and social conflict in bacteria. Phil Trans R Soc Lond B Biol Sci. 2015;370: 20140298. 26150664 10.1098/rstb.2014.0298 PMC4528496

[5] Cordero OX, Datta MS. Microbial interactions and community assembly at microscales. Curr Opin Microbiol. 2016;31: 227–234. 10.1016/j.mib.2016.03.015 27232202 PMC5157693

[6] Li L, Ma Z. Testing the neutral theory of biodiversity with human microbiome datasets. Sci Rep. 2016; 6: 31448 10.1038/srep31448 27527985 PMC4985628

[7] García-Bayona L, Comstock LE. Bacterial antagonism in host-associated microbial communities. Science. 2018;361: pii: eaat2456. 10.1126/science.aat2456 30237322

[8] Zhang MM, Poulsen M, Currie CA. Symbiont recognition of mutualistic bacteria by *Acromyrmex* leaf-cutting ants. ISME J. 2007;1: 313–320. 10.1038/ismej.2007.41 18043642

[9] Scheuring I, Yu DW. How to assemble a beneficial microbiome in three easy steps. Ecol Lett. 2012;15: 1300–1307. 10.1111/j.1461-0248.2012.01853.x 22913725 PMC3507015

[10] Coyte KZ, Schluter J, Foster KR. The ecology of the microbiome: Networks, competition, and stability. Science. 2015;350: 663–666. 10.1126/science.aad2602 26542567

[11] Foster KR, Schluter J, Coyte KZ, Rakoff-Nahoum S. The evolution of the host microbiome as an ecosystem on a leash. Nature. 2017;548: 43–51. 10.1038/nature23292 28770836 PMC5749636

[12] Duarte A, Welch M, Swannack C, Wagner J, Kilner RM. Strategies for managing rival bacterial communities: Lessons from burying beetles. J Anim Ecol. 2018;87: 414–427. 10.1111/1365-2656.12725 28682460 PMC5836980

[13] Innocent T, Holmes N, Al Bassam M, Schiott M, Scheuring I, Wilkinson B et al Experimental demonstration that screening can enable the environmental recruitment of a defensive microbiome. bioRxiv 2018; 10.1101/375634.

[14] Green JL, Bohannan BJ, Whitaker RJ. Microbial biogeography: from taxonomy to traits. Science. 2008;320: 1039–1043. 10.1126/science.1153475 18497288

[15] Jeraldo P, Sipos M, Chia N, Brulc JM, Dhillon AS, Konkel ME et al Quantification of the relative roles of niche and neutral processes in structuring gastrointestinal microbiomes. Proc Natl Acad Sci U S A. 2012;109: 9692–9698. 10.1073/pnas.1206721109 22615407 PMC3382495

[16] Fondi M, Karkman A, Tamminen MV, Bosi E, Virta M, Fani R et al “Every gene is everywhere but the environment selects”: Global geolocalization of gene sharing in environmental samples through network analysis. Genome Biol Evol. 2016;8: 1388–1400. 10.1093/gbe/evw077 27190206 PMC4898794

[17] Engl T, Kroiss J, Kai M, Nechitaylo TY, Svatoš A, Kaltenpoth M. Evolutionary stability of antibiotic protection in a defensive symbiosis. Proc Natl Acad Sci U S A. 2018;115: E2020–E2029. 10.1073/pnas.1719797115 29444867 PMC5834716

[18] West SA, Diggle SP, Buckling A, Gardner A, Griffin AS. The social lives of microbes. Annu Rev Ecol Evol Syst. 2007;38: 53–77.

[19] Bull JJ, Rice WR. Distinguishing mechanisms for the evolution of co-operation. J Theor Biol. 1991;149: 63–74. 1881147 10.1016/s0022-5193(05)80072-4

[20] Herre EA, Knowlton N, Mueller UG, Rehner SA. The evolution of mutualisms: exploring the paths between conflict and cooperation. Trends Ecol Evol. 1999;14: 49–53. 10234251 10.1016/s0169-5347(98)01529-8

[21] Sachs JL, Mueller UG, Wilcox TP, Bull JJ. The evolution of cooperation. Q Rev Biol. 2004;79: 135–160. 15232949 10.1086/383541

[22] Ebert D. The epidemiology and evolution of symbionts with mixed-mode transmission. Ann Rev Ecol Evol Syst. 2013;44: 623–643.

[23] Clay K. Defensive symbiosis: a microbial perspective. Funct Ecol. 2014;228: 293–298.

[24] Frank S. Host-symbiont conflict over the mixing of symbiotic lineages. Proc R Soc Lond B Biol Sci. 1996;263: 339–344. 10.1098/rspb.1996.0052 8920255

[25] Marsh SE, Poulsen M, Pinto-Tomás A, Currie CR. Interaction between workers during a short time window is required for bacterial symbiont transmission in *Acromyrmex* leaf-cutting ants. PLoS ONE. 2014;9: e103269 10.1371/journal.pone.0103269 25058579 PMC4110003

[26] Currie CR, Poulsen M, Mendenhall J, Boomsma JJ, Billen J. Coevolved crypts and exocrine glands support mutualistic bacteria in fungus-growing ants. Science. 2006;311: 81–83. 10.1126/science.1119744 16400148

[27] Li H, Sosa-Calvo J, Horn HA, Pupo MT, Clardy J, Rabeling C et al Convergent evolution of complex structures for ant–bacterial defensive symbiosis in fungus-farming ants. Proc Natl Acad Sci U S A. 2018; e-pub ahead of print 3 October 2018; 10.1073/pnas.1809332115 30282739 PMC6196509

[28] Kaltenpoth M, Göttler W, Herzner G, Strohm E. Symbiotic bacteria protect wasp larvae from fungal infestation. Curr Biol. 2005;15: 475–479. 10.1016/j.cub.2004.12.084 15753044

[29] Kaltenpoth M. *Actinobacteria* as mutualists: general healthcare for insects? Trends Microbiol. 2009;17: 529–535. 10.1016/j.tim.2009.09.006 19853457

[30] Seipke RF, Kaltenpoth M, Hutchings MI. *Streptomyces* as symbionts: an emerging and widespread theme? FEMS Microbiol Rev. 2012;36: 862–876. 10.1111/j.1574-6976.2011.00313.x 22091965

[31] Kroiss J, Kaltenpoth M, Schneider B, Schwinger MG, Hertweck C, Maddula RK et al Symbiotic *Streptomycetes* provide antibiotic combination prophylaxis for wasp offspring. Nat Chem Biol. 2010;6: 261–263. 10.1038/nchembio.331 20190763

[32] O’Callaghan M. Microbial inoculation of seed for improved crop performance: issues and opportunities. Appl Microbiol Biotechnol. 2016;100: 5729–5746. 10.1007/s00253-016-7590-9 27188775 PMC4909795

[33] Deaker R, Roughley RJ, Kennedy IR. Legume seed inoculation technology–a review. Soil Biol Biochem. 2004;36: 1275–1288.

[34] Werner GDA, Kiers ET. Order of arrival structures arbuscular mycorrhizal colonization of plants. New Phytol. 2014;205: 1515–1524. 10.1111/nph.13092 25298030

[35] Vojvodic S, Rehan SM, Anderson KE. Microbial gut diversity of Africanized and European honey bee larval instars. PLoS ONE. 2013;8: e72106 10.1371/journal.pone.0072106 23991051 PMC3749107

[36] Powell JE, Martinson VG, Urban-Mead K, Moran NA. Routes of acquisition of the gut microbiota of *Apis mellifera*. Appl Environ Microbiol. 2014;80: 7378–7387. 10.1128/AEM.01861-14 25239900 PMC4249178

[37] Schwarz RS, Moran NA, Evans JD. Early gut colonizers shape parasite susceptibility and microbiota composition in honey bee workers. Proc Natl Acad Sci U S A. 2016;113: 9345–9350. 10.1073/pnas.1606631113 27482088 PMC4995961

[38] Kwong WK, Moran NA. Gut microbial communities of social bees. Nat Rev Microbiol. 2016;14: 374–384. 10.1038/nrmicro.2016.43 27140688 PMC5648345

[39] Andersen SB, Yek SH, Nash DR, Boomsma JJ. Interaction specificity between leaf-cutting ants and vertically transmitted *Pseudonocardia* bacteria. BMC Evol Biol. 2015;15: 27 10.1186/s12862-015-0308-2 25886448 PMC4346108

[40] Martínez-García Á, Martín-Vivaldi M, Rodríguez-Ruano SM, Peralta-Sánchez JM, Valdivia E, Soler JJ. Nest bacterial environment affects microbiome of hoopoe eggshells, but not that of the uropygial secretion. PLoS ONE. 2016;11: e0158158 10.1371/journal.pone.0158158 27409772 PMC4943718

[41] Truyens S, Weyens N, Cuypers A, Vangronsveld J. Changes in the population of seed bacteria of transgenerationally Cd-exposed *Arabidopsis thaliana*. Plant Biol. 2012;15: 971–981. 10.1111/j.1438-8677.2012.00711.x 23252960

[42] Turnbaugh PJ, Hamady M, Yatsunenko T, Cantarel BL, Duncan A, Ley RE et al A core gut microbiome in obese and lean twins. Nature. 2009;457: 480–484. 10.1038/nature07540 19043404 PMC2677729

[43] Fukatsu T and Hosokawa T. Capsule-transmitted gut symbiotic bacterium of the Japanese common plataspid stinkbug, *Megacopta punctatissima*. Appl Environ Microbiol. 2002;68: 389–96. 10.1128/AEM.68.1.389-396.2002 11772649 PMC126591

[44] Hosokawa T, Kikuchi Y, Meng XY, Fukatsu T. he making of symbiont capsule in the plataspid stinkbug *Megacopta punctatissima*. FEMS Microbiol Ecol. 2005;54: 471–477. 10.1016/j.femsec.2005.06.002 16332344

[45] Hosokawa T, Hironaka M, Inadomi K, Mukai H, Nikoh N, Fukatsu T. Diverse strategies for vertical symbiont transmission among subsocial stinkbugs. PLoS One. 2013;8: e65081 10.1371/journal.pone.0065081 23741463 PMC3669201

[46] Bais HP, Weir TL, Perry LG, Gilroy S, Vivanco JM. The role of root exudates in rhizosphere interactions with plants and other organisms. Annu Rev Plant Biol. 2006;57: 233–66. 10.1146/annurev.arplant.57.032905.105159 16669762

[47] Hartmann A, Schmid M, van Tuinen D, Berg G. Plant-driven selection of microbes. Plant Soil. 2008;321: 235–257.

[48] Neal AL, Ahmad S, Gordon-Weeks R, Ton J. Benzoxazinoids in root exudates of maize attract *Pseudomonas putida* to the rhizosphere. PLoS ONE. 2012;7: e35498 10.1371/journal.pone.0035498 22545111 PMC3335876

[49] De Coninck B, Timmermans P, Vos C, Cammue BP, Kazan K. What lies beneath: belowground defense strategies in plants. Trends Plant Sci. 2015;20: 91–101. 10.1016/j.tplants.2014.09.007 25307784

[50] Cai T, Cai W, Zhang J, Zheng H, Tsou AM, Xiao L et al Host legume-exuded antimetabolites optimize the symbiotic rhizosphere. Mol Microbiol. 2009;73: 507–517. 10.1111/j.1365-2958.2009.06790.x 19602148

[51] Fang FC. Antimicrobial reactive oxygen and nitrogen species: concepts and controversies. Nat Rev Microbiol. 2004;2: 820–832. 10.1038/nrmicro1004 15378046

[52] Wang Y, Ruby EG. The roles of NO in microbial symbioses. Cell Microbiol. 2011;13: 518–526. 10.1111/j.1462-5822.2011.01576.x 21338463 PMC3690197

[53] Mandel MJ, Dunn AK. Impact and influence of the natural vibrio-squid symbiosis in understanding bacterial-animal interactions. Front Microbiol. 2016;15: 1982. 10.3389/fmicb.2016.01982 PMC5156696 28018314

[54] Davidson SK, Koropatnick TA, Kossmehl R, Sycuro L, McFall-Ngai MJ. NO means 'yes' in the squid-vibrio symbiosis: nitric oxide (NO) during the initial stages of a beneficial association. Cell Microbiol. 2004;6: 1139–1151. 10.1111/j.1462-5822.2004.00429.x 15527494

[55] Ruby EG, McFall-Ngai MJ. Oxygen-utilizing reactions and symbiotic colonization of the squid light organ by *Vibrio fischeri*. Trends Microbiol. 1999;7: 414–420. 10498950 10.1016/s0966-842x(99)01588-7

[56] Poole RK, & Hughes MN. New functions for the ancient globin family: bacterial responses to nitric oxide and nitrosative stress. Mol Microbiol. 2000;36: 775–783. 10844666 10.1046/j.1365-2958.2000.01889.x

[57] Wang Y, Dufour YS, Carlson HK, Donohue TJ, Marletta MA, Ruby EG. H-NOX-mediated nitric oxide sensing modulates symbiotic colonization by *Vibrio fischeri*. Proc Natl Acad Sci U S A. 2010;107: 8375–8380. 10.1073/pnas.1003571107 20404170 PMC2889544

[58] Wang Y, Dunn AK, Wilneff J, McFall-Ngai MJ, Spiro S, Ruby EG. *Vibrio fischeri* flavohaemoglobin protects against nitric oxide during initiation of the squid–*Vibrio* symbiosis. Mol Microbiol. 2010;78: 903–915. 10.1111/j.1365-2958.2010.07376.x 20815823 PMC2978254

[59] Franzenburg S, Walter J, Künzel S, Wang J, Baines JF, Bosch TCG et al Distinct antimicrobial peptide expression determines host species-specific bacterial associations. Proc Natl Acad Sci U S A. 2013;110: E3730–E3738. 10.1073/pnas.1304960110 24003149 PMC3785777

[60] Pietschke C, Treitz C, Forêt S, Schultze A, Künzel S, Tholey A et al Host modification of a bacterial quorum-sensing signal induces a phenotypic switch in bacterial symbionts. Proc Natl Acad Sci U S A. 2017;114: E8488–E8497. 10.1073/pnas.1706879114 28923926 PMC5635886

[61] Bernier SP, Surette MG. Concentration-dependent activity of antibiotics in natural environments. Front Microbiol. 2013;13: 20. 10.3389/fmicb.2013.00020 PMC3574975 23422936

[62] Schluter J, Nadell CD, Bassler BL, Foster KR. Adhesion as a weapon in microbial competition. ISME J. 2015;9: 139–149. 10.1038/ismej.2014.174 25290505 PMC4268496

[63] McLoughlin K, Schluter J, Rakoff-Nahoum S, Smith AL, Foster KR. Host selection of microbiota via differential adhesion. Cell Host Microbe. 2016;19: 550–559. 10.1016/j.chom.2016.02.021 27053168

[64] Raina J-B, Dinsdale EA, Willis BL, Bourne DG. Do the organic sulfur compounds DMSP and DMS drive coral microbial associations. Trends Microbiol. 2010;18: 101–108. 10.1016/j.tim.2009.12.002 20045332

[65] Raina J-B, Tapiolas D, Motti CA, Foret S, Seemann T, Tebben J et al Isolation of an antimicrobial compound produced by bacteria associated with reef-building corals. PeerJ. 2016;18: e2275. 10.7717/peerj.2275 PMC4994080 27602265

[66] Raina J-B, Tapiolas DM, Foret S, Lutz A, Abrego D, Ceh J et al DMSP biosynthesis by an animal and its role in coral thermal stress response. Nature. 2013;502: 677–680. 10.1038/nature12677 24153189

[67] Raina J-B, Tapiolas DM, Willis BL, Bourne DG. Coral-associated bacteria and their role in the biogeochemical cycling of sulfur. Appl Environ Microbiol. 2009;75: 3492–3501. 10.1128/AEM.02567-08 19346350 PMC2687302

[68] Apprill A, Marlow HQ, Martindale MQ, Rappé MS. The onset of microbial associations in the coral *Pocillopora meandrina*. ISME J. 2009;3: 685–699. 10.1038/ismej.2009.3 19242535

[69] Zivkovic AM, German JB, Lebrilla CB, Mills DA. Human milk glycobiome and its impact on the infant gastrointestinal microbiota. Proc Natl Acad Sci U S A. 2011;108: 4653–4658. 10.1073/pnas.1000083107 20679197 PMC3063602

[70] Haichar FZ, Marol C, Berge O, Rangel-Castro JI, Prosser JI, Balesdent J et al Plant host habitat and root exudates shape soil bacterial community structure. ISME J. 2008;2: 1221–1230. 10.1038/ismej.2008.80 18754043

[71] Badri DV, Vivanco JM. Regulation and function of root exudates. Plant Cell Environ. 2009;32: 666–681. 10.1111/j.1365-3040.2008.01926.x 19143988

[72] Dennis PG, Miller AJ, Hirsch PR. Are root exudates more important than other sources of rhizodeposits in structuring rhizosphere bacterial communities? FEMS Microbiol Ecol. 2010;72: 313–327. 10.1111/j.1574-6941.2010.00860.x 20370828

[73] Badri DV, Chaparro JM, Zhang R, Shen Q, Vivanco JM. Application of natural blends of phytochemicals derived from the root exudates of *Arabidopsis* to the soil reveal that phenolic-related compounds predominantly modulate the soil microbiome. J Biol Chem. 2013;288: 4502–4512. 10.1074/jbc.M112.433300 23293028 PMC3576057

[74] Lebeis SL, Paredes SH, Lundberg DS, Breakfield N, Gehring J, McDonald M et al Salicylic acid modulates colonization of the root microbiome by specific bacterial taxa. Science. 2015;349: 860–864. 10.1126/science.aaa8764 26184915

[75] Ishiyama D, Vujaklija D, Davies J. Novel pathway of salicylate degradation by *Streptomyces sp*. strain WA46. Appl Environ Microbiol. 2004;70: 1297–1306. 10.1128/AEM.70.3.1297-1306.2004 15006746 PMC368302

[76] Wright GD. Mechanisms of resistance to antibiotics. Curr Opin Chem Biol 2003;7: 563–569. 14580559 10.1016/j.cbpa.2003.08.004

[77] Ghoul M, Mitri S. The ecology and evolution of microbial competition. Trends Microbiol. 2016;24: 833–845. 10.1016/j.tim.2016.06.011 27546832

[78] Raynaud X, Nunan N. Spatial ecology of bacteria at the microscale in soil. PLoS ONE. 2014;9: e87217 10.1371/journal.pone.0087217 24489873 PMC3905020

[79] Levins R, Culver D. Regional coexistence of species and competition between rare species. Proc Natl Acad Sci U S A. 1971;68: 1246–1248. 10.1073/pnas.68.6.1246 16591932 PMC389163

[80] Nee S, May RM. Dynamics of metapopulations: habitat destruction and competitive coexistence. J Anim Ecol. 1992;61: 37.

[81] Yu DW, Wilson HB. The competition-colonization trade-off is dead; long live the competition-colonization trade-off. Am Nat. 2001;158: 49–63. 10.1086/320865 18707314

[82] Tilman D. Competition and biodiversity in spatially structured habitats. Ecology. 1994;75: 2–16.

[83] Wright GD. Bacterial resistance to antibiotics: enzymatic degradation and modification. Adv Drug Deliv Rev. 2005;57: 1451–1470. 10.1016/j.addr.2005.04.002 15950313

[84] Kumar A, Schweizer HP. Bacterial resistance to antibiotics: active efflux and reduced uptake. Adv Drug Deliv Rev. 2005;57: 1486–513. 10.1016/j.addr.2005.04.004 15939505

[85] Marquez B. Bacterial efflux systems and efflux pumps inhibitors. Biochimie. 2005;87: 1137–1147. 10.1016/j.biochi.2005.04.012 15951096

[86] Davies J, Davies D. Origins and evolution of antibiotic resistance. Microbiol Mol Biol Rev. 2010;74: 417–433. 10.1128/MMBR.00016-10 20805405 PMC2937522

[87] Kondrat S, Zimmermann O, Wiechert W, von Lieres E. Discrete-continuous reaction-diffusion model with mobile point-like sources and sinks. Eur Phys J E. 2016;39: 11 10.1140/epje/i2016-16011-0 26830760

[88] Rodriguez R, Redman R. More than 400 million years of evolution and some plants still can't make it on their own: plant stress tolerance via fungal symbiosis. J Exp Bot. 2008;59: 1109–1114. 10.1093/jxb/erm342 18267941

[89] Lau JA, Lennon JT. Evolutionary ecology of plant–microbe interactions: soil microbial structure alters selection on plant traits. New Phytol. 2011;192: 215–224. 10.1111/j.1469-8137.2011.03790.x 21658184

[90] Rolli E, Marasco R, Vigani G, Ettoumi B, Mapelli F, Deangelis ML et al Improved plant resistance to drought is promoted by the root-associated microbiome as a water stress-dependent trait. Environ Microbiol. 2014;17: 316–31. 10.1111/1462-2920.12439 24571749

[91] Wang ZK, Yang YS, Stefka AT, Sun G, Peng LH. Review article: fungal microbiota and digestive diseases. Aliment Pharmacol Ther. 2014;39: 751–766. 10.1111/apt.12665 24612332

[92] Mueller UG, Sachs JL. Engineering microbiomes to improve plant and animal health. Trends Ecol Evol. 2015;23: 606–617. 10.1016/j.tim.2015.07.009 26422463

[93] Shreiner AB, Kao JY, Young VB. The gut microbiome in health and in disease. Curr Opin Gastroenterol. 2015;31: 69–75. 10.1097/MOG.0000000000000139 25394236 PMC4290017

[94] Lloyd-Price J, Abu-Ali G, Huttenhower C. The healthy human microbiome. Genome Med. 2016;8: 51 10.1186/s13073-016-0307-y 27122046 PMC4848870

[95] Lloyd DP, Allen RJ. Competition for space during bacterial colonization of a surface. J R Soc Interface. 2015;12: 20150608 10.1098/rsif.2015.0608 26333814 PMC4614474

[96] Haichar FZ, Santaella C, Heulin T, Achouak W. Root exudates mediated interactions belowground. Soil Biol Biochem. 2014;77: 69–80.

[97] Wright GD. Antibiotic resistance in the environment: a link to the clinic? Curr Opin Microbiol. 2010;13: 589–594. 10.1016/j.mib.2010.08.005 20850375

[98] Harcombe WR, Riehl WJ, Dukovski I, Granger BR, Betts A, Lang AH et al Metabolic resource allocation in individual microbes determines ecosystem interactions and spatial dynamics. Cell Metab. 2014;7: 1104–1115. 10.1016/j.celrep.2014.03.070 PMC4097880 24794435

