# Supplementary figures and images for: Efficient assembly and long-term stability of defensive microbiomes via private resources and community bistability
Source: PLoS Comput Biol. 2019 May 31;15(5):e1007109. doi: 10.1371/journal.pcbi.1007109 (PMC6576795; doi:10.1371/journal.pcbi.1007109)

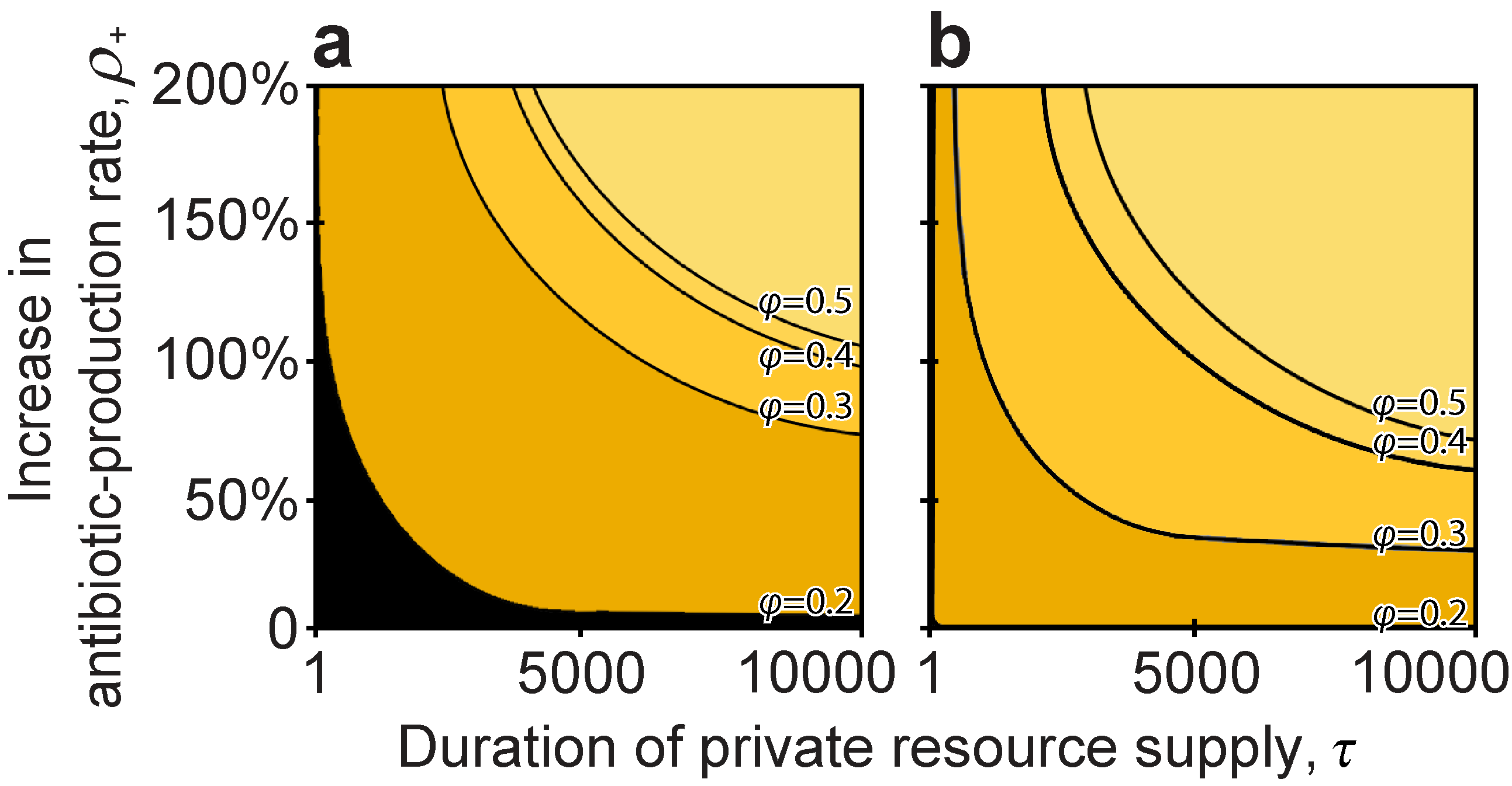

Supplement: S1 Fig — High extracellular decay rates reduce the effectiveness of the antibiotic and thus decrease the effectiveness of the help provided by the host to the beneficial microbe for τ time. Therefore, to compensate for higher decay rates, higher effort is needed from the host either in the form of more help (along the y-axis) or in the form of same amount of help provided for longer time (x-axis). Results are shown for (a) zero (βB = 0) and for (b) modest (βB = 0.25) efflux rates. The upper right areas correspond to beneficial-dominated outcomes, while the bottom left corners correspond to parasite-dominated outcomes. The light (ochre) shadings represent the regions in which the beneficial strain wins for more than 50% of the simulations, and the darker (black) shaded areas mark the parameter combinations in which the parasitic strain wins for the majority of simulations. The black lines mark the boundaries separating these two regions. Model parameters are: rB,0 = 0.8, rP,0 = 0.8, c = 0.1, ρB,0 = 1, αB = 0.5, αP = 0.5, βP = 0, γB = 0.4, γP = 0.4, D = 5, a = 1, T = 1, k = 25, N = 10 000, nB,0 = 100, nP,t = 10, κ = 1, f = 0.01, Δt = 1/10, u = 100, r+ = 0, and s+ = 0. (TIF) [file pcbi.1007109.s001.tif]

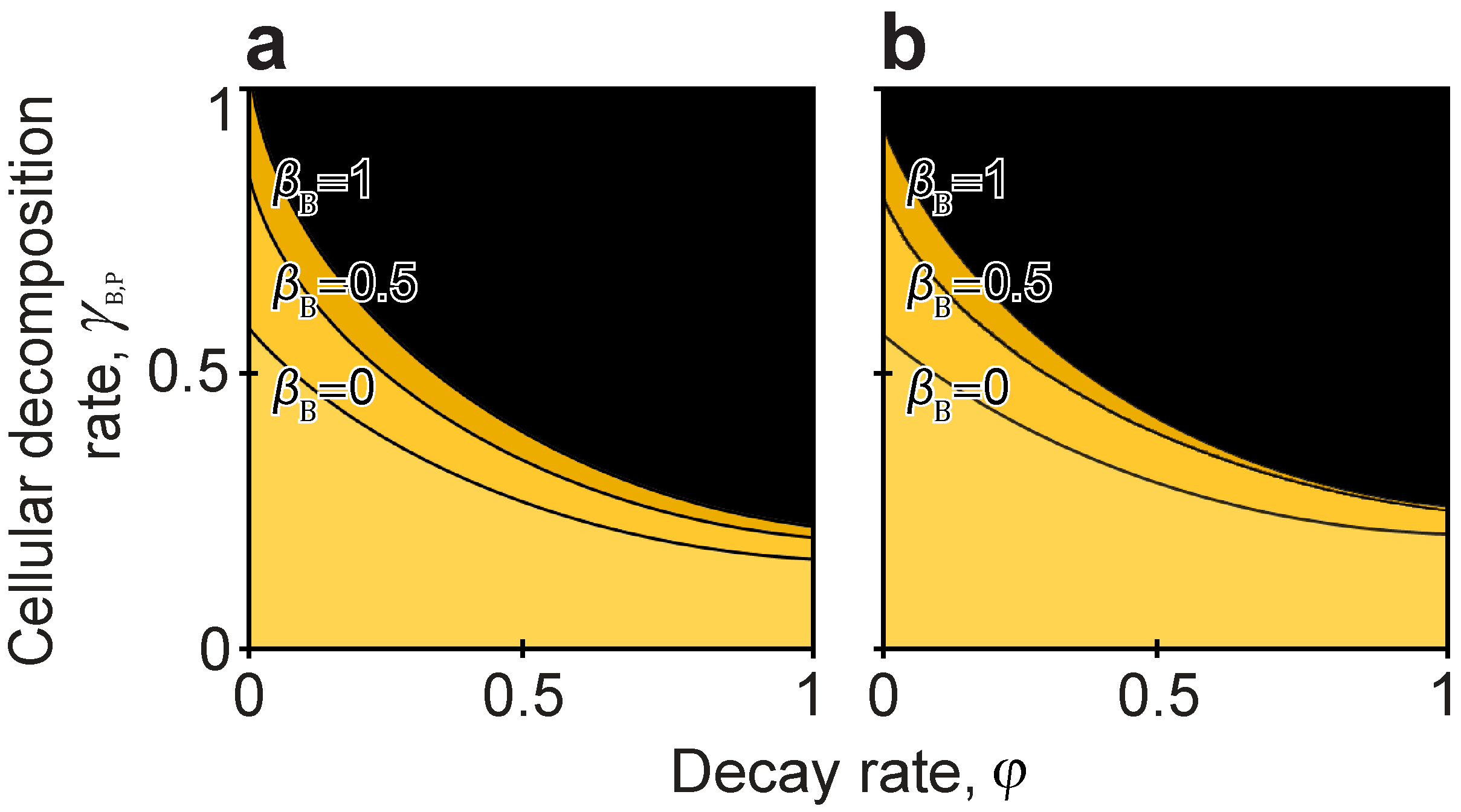

Supplement: S2 Fig — High decay rates reduce the effect of the antibiotic; hence, the antibiotic is effective against the parasitic strain only if the decomposition rate is low and the antibiotic accumulates more quickly in the sensitive cells to lethal concentrations. Results are shown for (a) low (D = 0.5) and (b) modest (D = 5) diffusion rates. The upper right areas correspond to parasite-dominated outcomes, and the bottom left areas correspond to beneficial-dominated outcomes. The light (ochre) shadings represent the regions in which the beneficial strain wins for more than 50% of the simulations, and the darker (black) shaded areas mark the parameter combinations in which the parasitic strain wins for the majority of simulations. The black lines mark the boundaries separating these two regions. Model parameters are: rB,0 = 0.8, rP,0 = 0.8, c = 0.1, ρB,0 = 1, αB = 0.5, αP = 0.5, βP = 0, a = 1, T = 1, k = 25, N = 10 000, nB,0 = 100, nP,t = 10, κ = 1, f = 0.01, Δt = 1/10, u = 100, τ = 0, r+ = 0, s+ = 0, and ρ+ = 0. (TIF) [file pcbi.1007109.s002.tif]

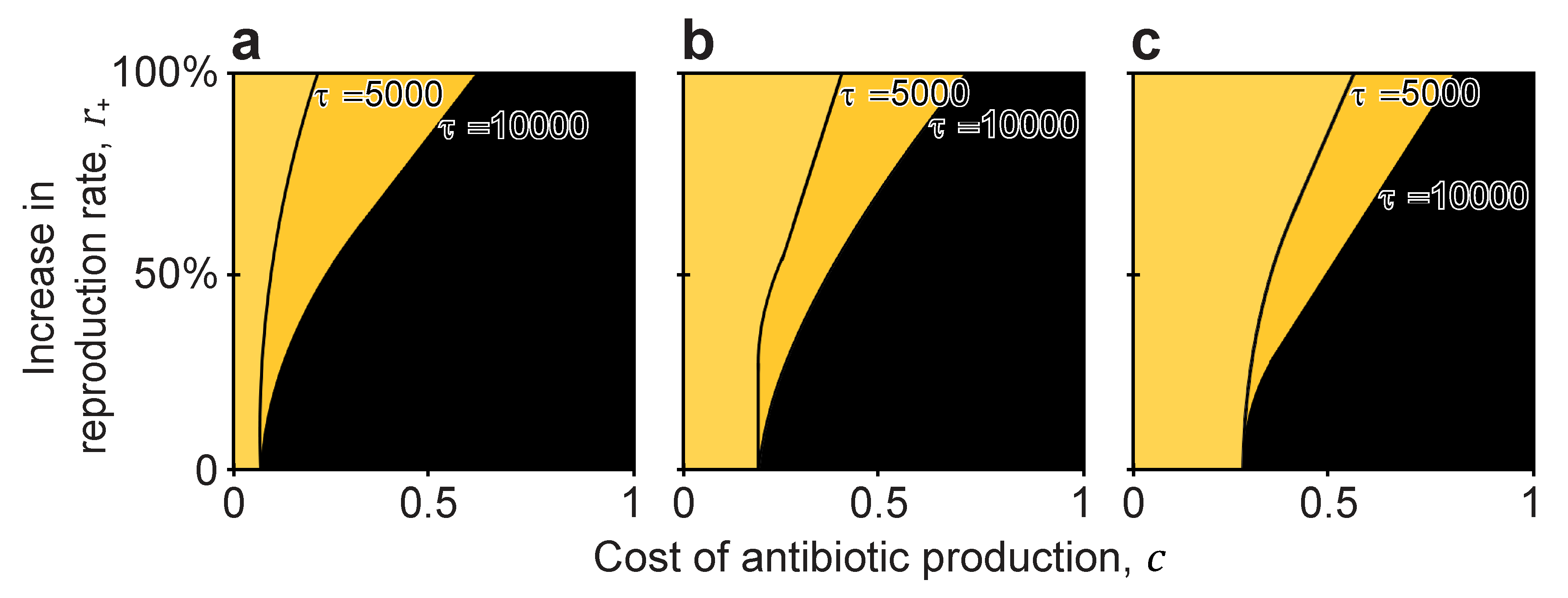

Supplement: S3 Fig — The higher the cost of producing the antibiotic (x-axis), the more support from the host, in the form of private resource increasing the growth rate of the beneficial (y-axis) is required to secure the dominance of the beneficial strain. The longer that the private resource is provided (τ) the higher the cost that can be tolerated. Results are shown for (a) low (βB = 0), (b) modest (βB = 0.25), and (c) medium (βB = 0.5) efflux rates. The upper left areas correspond to beneficial-dominated outcomes, while the right-hand and bottom right areas correspond to parasite-dominated outcomes. The light (ochre) shadings represent the regions in which the beneficial strain wins for more than 50% of the simulations, and the darker (black) shaded areas mark the parameter combinations in which the parasitic strain wins for the majority of simulations. The black lines mark the boundaries separating these two regions. Model parameters are: rB,0 = 0.8, rP,0 = 0.8, ρB,0 = 1, αB = 0.5, αP = 0.5, βP = 0, γB = 0.4, γP = 0.4, φ = 0.3, D = 5, a = 1, T = 1, k = 25, N = 10 000, nB,0 = 100, nP,t = 10, κ = 1, f = 0.01, Δt = 1/10, u = 100, s+ = 0, and ρ+ = 0. (TIF) [file pcbi.1007109.s003.tif]

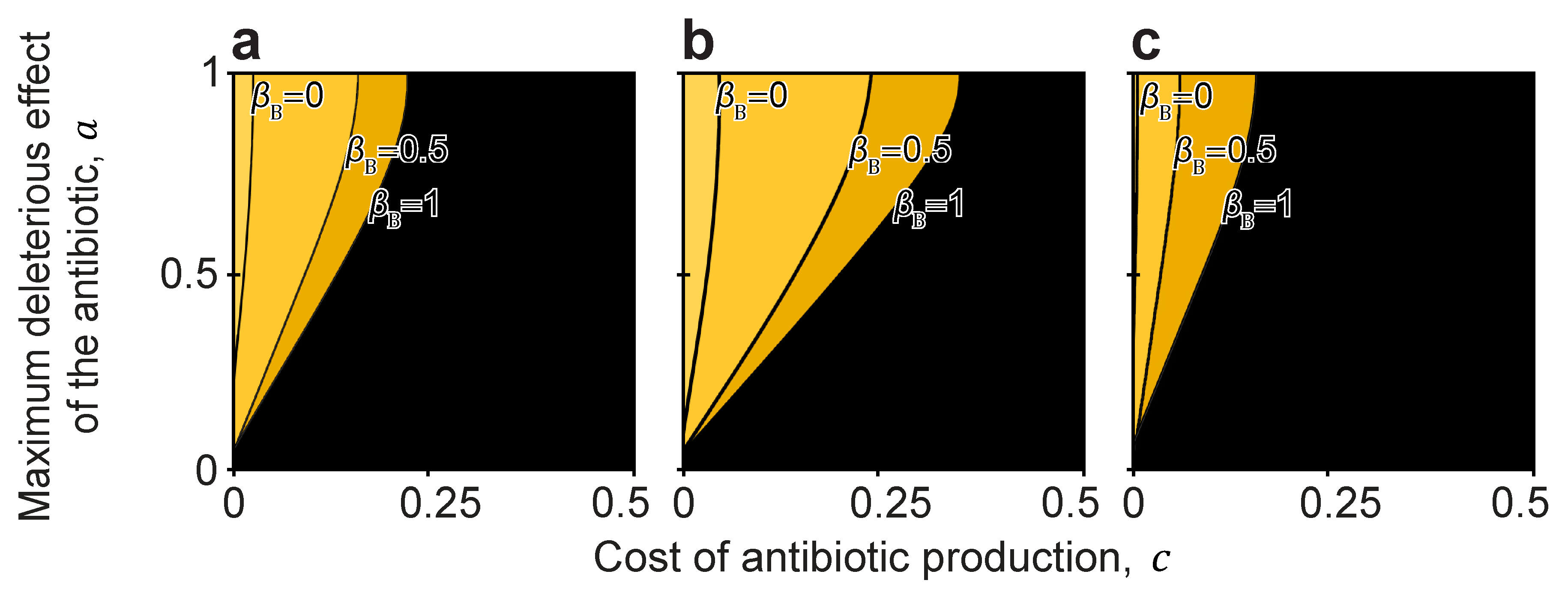

Supplement: S4 Fig — The higher the cost of producing the antibiotic (x-axis), the more effective the antibiotic (y-axis) must be to secure the dominance of the beneficial strain (where effectiveness is measured as reducing the growth rate of the parasitic strain). Also, the higher the efflux rate (β), the larger the cost can be tolerated. Very low (a; D = 0.5) and very high (c; D = 50) diffusion rates hinder the effectiveness of the antibiotic, hence the highest costs are tolerated at medium (b; D = 5) diffusion rates. The upper left corners correspond to beneficial-dominated outcomes, while the right-hand and bottom right areas correspond to parasite-dominated outcomes. The light (ochre) shadings represent the regions in which the beneficial strain wins for more than 50% of the simulations, and the darker (black) shaded areas mark the parameter combinations in which the parasitic strain wins for the majority of simulations. The black lines mark the boundaries separating these two regions. Model parameters are: rB,0 = 0.8, rP,0 = 0.8, ρB,0 = 1, αB = 0.5, αP = 0.5, βP = 0, γB = 0.4, γP = 0.4, φ = 0.3, T = 1, k = 25, N = 10 000, nB,0 = 100, nP,t = 10, κ = 1, f = 0.01, Δt = 1/10, u = 100, τ = 2000, r+ = 0.4, s+ = 0, and ρ+ = 0. (TIF) [file pcbi.1007109.s004.tif]

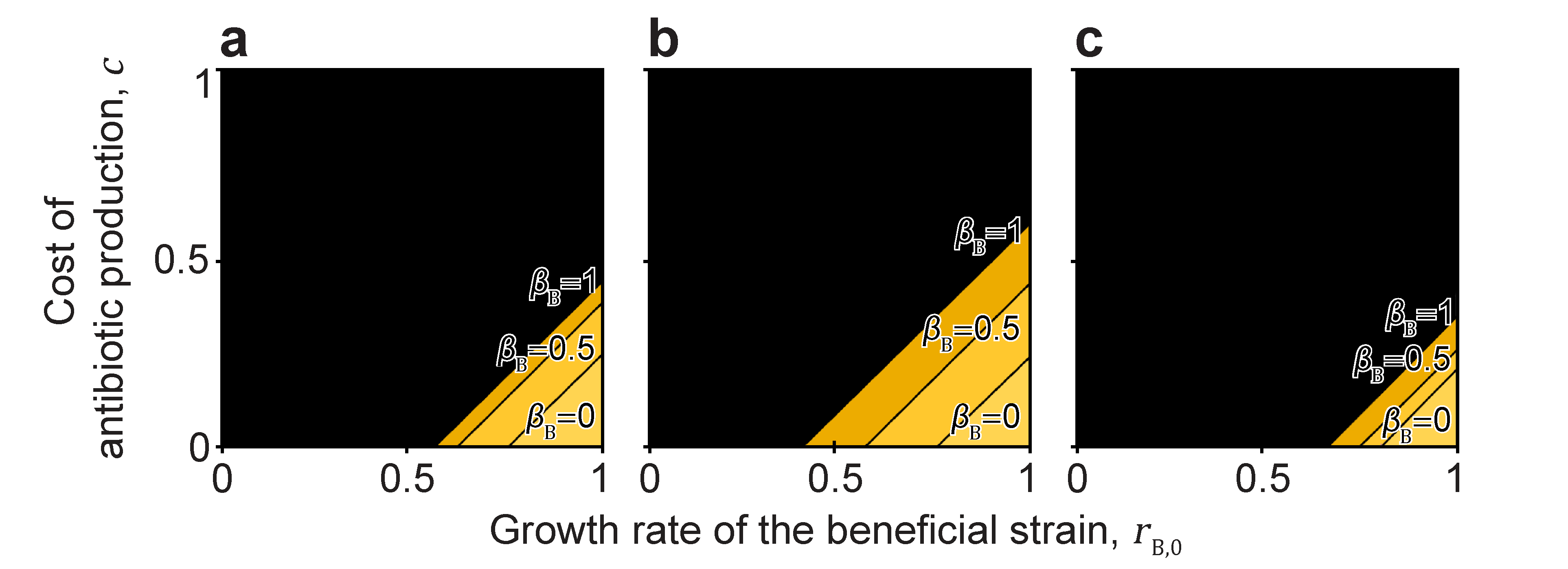

Supplement: S5 Fig — High reproduction rates allow for higher costs, as the competitive disadvantage caused by the costly antibiotic production can be compensated by higher reproduction rates compared to that of the parasitic strain’s (rP,0 = 0.8). Higher efflux rates (βB = 0 → βB = 1) can also help to compensate for a competitive disadvantage caused by lower reproduction rates and costly antibiotic production. Results are shown for (a) low (D = 0.5), (b) medium (D = 5), and (c) high (D = 50) diffusion rates. Increasing the diffusion rate further improves the effectiveness of the antibiotic, and hence the beneficial can dominate even with relatively high cost and low reproduction rates, until a certain point. Very high diffusion rates, on the other hand, hinder the effectiveness of the antibiotic. The upper left areas correspond to parasite-dominated outcomes, while the bottom right corners correspond to beneficial-dominated outcomes. The light (ochre) shadings represent the regions in which the beneficial strain wins for more than 50% of the simulations, and the darker (black) shaded areas mark the parameter combinations in which the parasitic strain wins for the majority of simulations. The black lines mark the boundaries separating these two regions. Model parameters are: rP,0 = 0.8, c = 0.1, ρB,0 = 1, αB = 0.5, αP = 0.5, βP = 0, γB = 0.4, γP = 0.4, φ = 0.3, D = 5, a = 1, T = 1, k = 25, N = 10 000, nB,0 = 100, nP,t = 10, κ = 1, f = 0.01, Δt = 1/10, u = 100, τ = 2000, r+ = 0.4, s+ = 0, and ρ+ = 0. (TIF) [file pcbi.1007109.s005.tif]

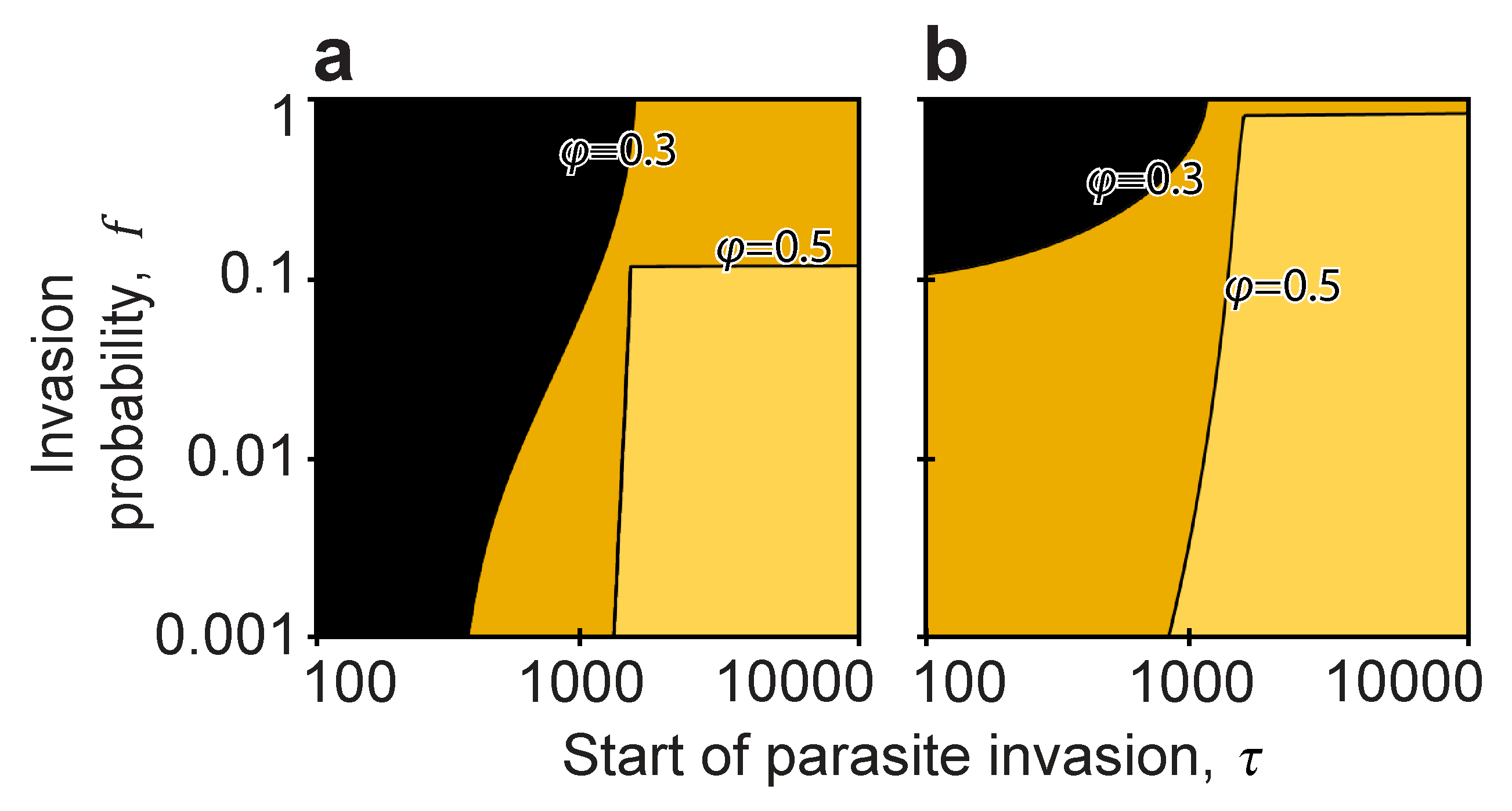

Supplement: S6 Fig — During these simulations, the invasion of the parasitic strain is blocked on the entire host surface, thus directing the host-provided resource entirely to the beneficial strain, until time τ (τ = κ). The higher the probability of invasion by the parasite (y-axis), the more time is necessary for the beneficial to reach a colony size big enough to resist invasion (x-axis). The higher the extracellular decay rate is (φ), the more time is required for the beneficial colony to become resistant to invasion, but this disadvantage can be compensated by high efflux rates (βB = 0 for a and βB = 0.25 for b). There is a limit, however, to how much invasion pressure the system can withstand; as can be seen, for high decay rates and above a certain invasion pressure (high f values along the y-axis), even long-lasting host support cannot result in dominance of the beneficial strain. The upper left corners correspond to parasite-dominated outcomes, while the bottom right areas correspond to beneficial-dominated outcomes. The light (ochre) shadings represent the regions in which the beneficial strain wins for more than 50% of the simulations, and the darker (black) shaded areas mark the parameter combinations in which the parasitic strain wins for the majority of simulations. The black lines mark the boundaries separating these two regions. Model parameters are: rB,0 = 0.8, rP,0 = 0.8, c = 0.1, ρB,0 = 1, αB = 0.5, αP = 0.5, βP = 0, γB = 0.4, γP = 0.4, D = 5, a = 1, T = 1, k = 25, N = 10 000, nB,0 = 100, nP,t = 10, Δt = 1/10, u = 100, r+ = 0, s+ = 1, and ρ+ = 0. (TIF) [file pcbi.1007109.s006.tif]
